# Multiplexed detection of respiratory virus RNA using optical pH sensors and injection-molded centrifugal microfluidics

**DOI:** 10.1007/s00604-025-06996-3

**Published:** 2025-02-12

**Authors:** Gianmarco Domenico Suarez, Yuki Yu Kiu Tang, Steevanson Bayer, Peter Pak-Hang Cheung, Stefan Nagl

**Affiliations:** 1 Hong Kong University of Science and Technology, Clear Water Bay, Kowloon, Hong Kong SAR; 2 Quommni Technologies, Tsuen Wan, New Territories Hong Kong SAR; 3 Department of Chemical Pathology, The Chinese University of Hong Kong, Shatin, New Territories Hong Kong SAR

**Keywords:** Isothermal amplification, Respiratory viruses, Fluorescent optical sensors, Centrifugal microfluidics, Injection molding

## Abstract

The application is demonstrated of injection-molded centrifugal microfluidic chips with integrated optical pH sensors for multiplexed detection of respiratory syndrome coronavirus 2 (SARS-CoV-2), influenza A, and influenza B RNA. The optical pH sensors generated sensitive fluorescent readouts from diagnostic reverse transcription loop-mediated isothermal amplification (RT-LAMP) reactions; limits of detection for influenzas A and B, and SARS-CoV-2 of 89, 245, and 38 RNA copies per reaction, respectively, were attained. Results were obtainable within 44 min for SARS-CoV-2 and influenza A, and 48 min for influenza B. We implemented a data processing strategy based on numerical derivatives of the fluorescence curves that allowed for reliable, quantitative thresholds for deciding reaction outcomes and enabled 100% specificity. This work demonstrates the utility of optical pH sensors and injection-molded centrifugal microfluidics for multiplexed infectious disease diagnostics with point-of-care applications.

**Graphical Abstract:**

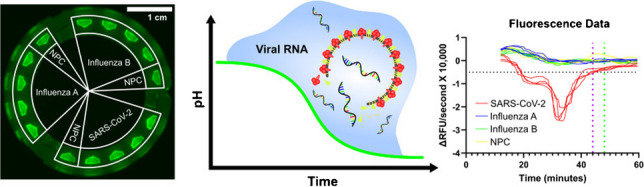

**Supplementary Information:**

The online version contains supplementary material available at 10.1007/s00604-025-06996-3.

## Introduction

Infectious diseases are illnesses caused by infectious agents, including bacteria, parasites, fungi, and viruses [1]. Infectious diseases are responsible for substantial morbidity and mortality worldwide: in 2019, they caused 420 million lost disability-adjusted life years and nearly 8 million deaths (over 10% of deaths globally) [2]. They are a continuing and growing problem. This can be attributed to, among others, the following factors: re-emergence of certain infectious diseases such as tuberculosis [3], antibiotic resistance in bacteria [4], the interplay between the necessity for expanding food production and disease emergence [5], and emerging infectious diseases of zoonotic origin [6]. The impact of infectious diseases is underscored by the coronavirus disease 2019 (COVID-19) pandemic, presumably caused by the zoonotic transmission of a novel coronavirus, severe acute respiratory syndrome coronavirus 2 (SARS-CoV-2) [7–9]. SARS-CoV-2 was first identified in January of 2020, and in March of 2020, a global pandemic was declared [10, 11]. Over 350,000 deaths worldwide were recorded by May of 2020, rising to over 6.5 million by October of 2022 [11, 12]. In addition to substantial loss of life, SARS-CoV-2 negatively impacted the world economy on a huge scale, highlighting the multi-faceted ways in which infectious diseases can harm society [13, 14]. Diagnosing and monitoring infectious diseases is therefore important, since it promotes better individual treatments and improves public health [15, 16].

The initial spread of SARS-CoV-2 can be partly attributed to a lack of efficacious interventional therapies available at the time [17, 18]. The lack of treatments consequently made routine testing and diagnosis of infected individuals critical to monitoring and preventing the virus’ transmission and spread, especially because of the prevalence of asymptomatic carriers [19, 20]. Hence, accurate and scalable diagnostic platforms were needed to mitigate the spread of SARS-CoV-2 and reduce mortalities. To achieve diagnostic capacity at the scale needed for containing future outbreaks, reliable and rapid point-of-care (POC) diagnostic methods are needed [21]. While an unbridled level of resources and scientific enquiry was devoted to characterizing and combating SARS-CoV-2, there are still other diseases, such as influenza, which pose challenges to public health [22, 23]. Therefore, emerging diagnostic technologies should also be multiplexed to screen multiple pathogens simultaneously [24].

Nucleic acid amplification tests (NAATs) are well-suited to the detection of infectious pathogens [25]. The polymerase chain reaction (PCR), for example, can exponentially amplify a small number of nucleic acid fragments specific to particular species or strains of pathogens, including bacteria and viruses [26]. Readouts from PCR-amplified products make it useful for infectious disease diagnostics, with exceptional sensitivity and specificity [27]. Reverse transcription quantitative PCR (RT-qPCR), a variant of PCR used for detecting RNA, has been able to detect SARS-CoV-2 in patients with sensitivities of approximately 80% and specificities of 100% [28, 29]. For comparison, rapid antigen tests (RATs) had much lower sensitivities ranging from 23 to 71% [30].

Furthermore, assay design is relatively straightforward for NAAT’s, requiring only the sequence being targeted and the production of oligonucleotides recognizing that sequence [31]. In contrast, RATs require the integration of antibodies, which can be more difficult to produce [32]. However, a key drawback to NAATs is that they generally require expensive machinery and relatively high levels of expertise to perform [33]. Therefore, PCR assays are typically performed in centralized laboratories, which can be a challenge in low-resource settings [24]. This can hamper public health and medical efforts to curtail outbreaks and treat patients, respectively. POC alternatives to gold standard methods are needed, enabled by newer technologies such as isothermal amplification and microfluidics [26, 34].

Microfluidic devices are characterized by their handling of minute liquid volumes using sub-millimeter sized structures, and have emerging applications in healthcare [35–37]. They can make it easier to perform diagnostic assays by automating the setup of reactions and reducing expenses by minimizing reagent usage [38]. A promising class of microfluidics, especially for POC applications, is centrifugal microfluidics. Centrifugal microfluidics utilize rotational forces (i.e., centrifugal, Coriolis and Euler forces) to move fluids through channels and into chambers [39, 40]. The integration of passive valves to regulate fluid flow simplifies the fabrication of centrifugal microfluidic chips, and metering chambers enable precise aliquoting and distribution of fluids to separate chambers [39]. These microfluidics at minimum require only a motor for actuation [41, 42].

Microfluidic chips enable multiplexing of diagnostic reactions [43, 44]. Isothermal amplification reactions, such as loop-mediated isothermal amplification (LAMP), are a promising alternative to PCR since they do not require thermocycling, making assay design technically simpler [45–47]. LAMP reactions can be performed on microfluidic chips and can detect DNA or RNA in the case of reverse transcription LAMP (RT-LAMP) [48, 49]. LAMP reactions confer microfluidic assays with adaptability since they use readily synthesized oligomer primers as the specific detection reagents [45, 46]. This means that diagnostic panels can be rapidly customized to detect a variety of emerging infectious agents and their variants. LAMP also generates substantial quantities of products and byproducts, which can be detected through various readout mechanisms [50, 51].

Multiple methods have been used to generate signals from LAMP reactions, detecting either the products (i.e., DNA) or byproducts (i.e., magnesium pyrophosphate and hydrogen ions) [47]. One method involves pH indicators, either colorimetric or fluorescent, to determine pH changes in minimally buffered LAMP reactions [47, 52, 53]. In another work, we built upon these methods by utilizing fluorescent optical pH sensors to generate readouts from LAMP reactions for detecting DNA fragments [54]. The optical pH sensors comprised fluorescein isothiocyanate (FITC) covalently immobilized within a poly(2-hydroxyethyl methacrylate) (pHEMA) matrix and were drop-casted as a precursor solution into microfluidic reaction chambers. Evaporation of the solvent yielded the complete fluorescent sensor layers.

Fluorescent optical pH sensors have excellent sensitivity and selectivity [55, 56], and are amenable to quantitative readouts, overcoming skewed color responses that can occur with colorimetric indicators [57, 58]. Additionally, fluorescent dyes exhibit high stability when integrated into a polymer matrix [59–61]. Fluorescent optical pH sensors have been utilized in microfluidic systems for cell culture [62–67], single cell experiments [68], enzymatic reaction monitoring [69–71], free-flow electrophoresis [72–75], and oral biofilm characterization [76], among other applications. In this study, we expand their application by utilizing them for RNA-based RT-LAMP diagnostic methods. We integrated optical pH sensors with centrifugal microfluidics, demonstrating a novel POC technology for distinguishing SARS-CoV-2, influenza A, and influenza B RNA via RT-LAMP on a single chip. The straightforward method of drop-casting the optical pH sensors onto the injection-molded microfluidic chips promotes the scalability of the technology.

**Fig. 1:**
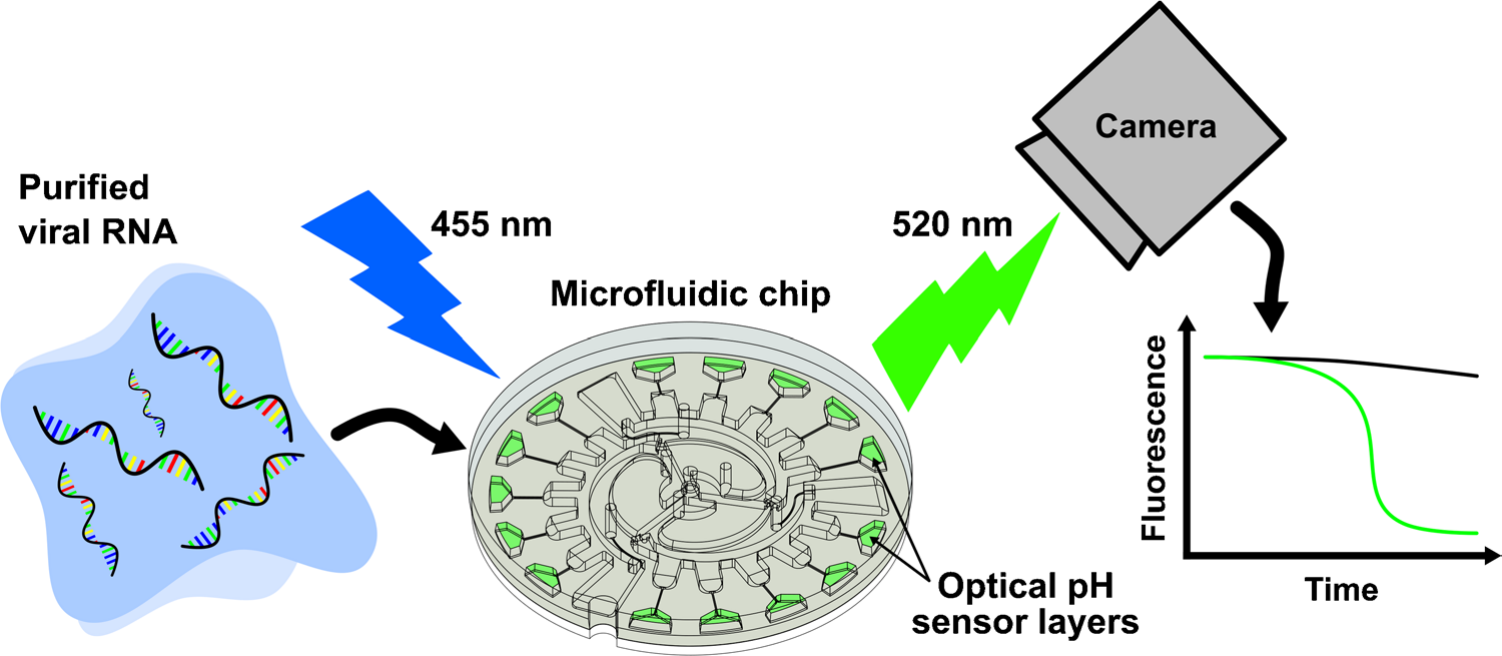
Schematic of diagnostics using a VDIM centrifugal microfluidic chip with integrated optical pH sensors. After loading viral RNA into the chip, it is actuated and then incubated. During RT-LAMP incubation, fluorescence from the optical pH sensors is imaged at regular intervals, and the images are processed into fluorescence curves. These curves are then analyzed to assess amplification and the presence of a specific viral RNA

The sensors’ quantitative, automated outputs yield good assay reliability by minimizing user bias and obviating special training. The chips were fabricated using variotherm desktop injection molding (VDIM), a technique bridging the gap between conventional prototyping and mass production methods [54]. It has a higher production rate than conventional prototyping methods with cycle times under 2 min and is cost-effective just like the optical pH sensor and its drop-casting-based microfluidic chip integration method (Supp. Table 1). Altogether the costs for each chip were at around 5 US $, which could potentially be reduced even further if the chips were mass produced. This work’s innovative technological platform demonstrates promising applications in clinical and domestic settings, where it could inform medical practitioners and laypersons of an infectious disease’s presence and substantially improve public health responses to future outbreaks (Fig. 1).

## Materials and methods

### Oligonucleotides

All oligonucleotides were from Integrated DNA Technologies (IDT, USA), desalted and resuspended at a concentration of 100 μM in water. 5 × LAMP primer master mixes were made comprising 8 μM FIP, 8 μM BIP, 1 μM F3, 1 μM B3, 2 μM LF, and 2 μM LB primers in nuclease-free ultrapure water (Beyotime, China).

### Optical pH sensor titration

Britton-Robinson buffers (BRBs) of varying pH levels were made with 40 mM H_3_BO_3_, H_3_PO_3_ and CH_3_COOH, respectively; pH was adjusted with 1 M NaOH. Fluorescein isothiocyanate (FITC, Sigma-Aldrich, USA) was covalently linked to poly-(2-hydroxyethyl methacrylate) (pHEMA, Sigma-Aldrich, USA) to make FITC-pHEMA optical pH probes as described before by Herzog et al. [72]. Briefly, 500 mg of pHEMA was dissolved in 5 mL of N,N-dimethylacetamide (DMAA) and 0.8 mg of FITC was added. The mixture was stirred for 3 h at 75 °C and then cooled to room temperature. FITC-pHEMA was then precipitated with BRB pH 6.0 and washed three times with the same buffer. A 5% (w/w) precursor solution was made by dissolving the product in ethanol/water (9:1 v/v) by stirring for 4 h at room temperature, before being stored at 4 °C.

For the optical pH sensor titration, the precursor solution was diluted to 40% in water and 25 µL was drop-casted into separate wells of a 96-well black polystyrene plate (Greiner Bio-One, Germany). The plates were heated in an oven at 70 °C for 10 min. The sensor spots were then covered with 150 µL of BRBs with varying pH levels. Fluorescent readings were taken for each well using a FlexStation 3 Multi-mode Microplate Reader (Molecular Devices, USA), with excitation at 490 nm and emission readings at 525 nm.

### Microfluidic chip fabrication

Centrifugal microfluidic chips comprised two halves: an injection-molded top with microfluidic features and a CNC-milled bottom layered with a double-sided pressure sensitive adhesive (PSA). The injection-molded upper halves were made in-house using an HJK-12 T (Haijiang, China) injection molding machine and PG-383 polystyrene pellets (Chimei, China). The design, fabrication, and use of injection molds have been described more thoroughly elsewhere [54]. The bottom halves were made by layering a double-sided PSA (No. 5605, Nitto, Japan) onto a 0.75-mm-thick white polystyrene sheet (Evergreen Scale Models, United States) and cutting the sheet into pieces of approx. 40 mm by 50 mm in size. A CNC4030-2.2 kW milling machine (Jingyan Instruments, China) was then used to cut these pieces into the shape of the microfluidic halves.

Microfluidic chip halves were post-processed with a CNC-mill and cleaned. Into each of the reaction chambers, 3 μL of FITC-pHEMA sensor precursor solution was drop-casted and dried at 70 °C in an oven for 10 min. Once dry, any excess sensor layer on the periphery of the chambers or in the adjoining capillary valves was scraped away with a wet sterile toothpick. Primer mix was then deposited in the appropriate reaction chambers and dried at 35 °C on a hot plate for 30 min. Two microliters of SARS-CoV-2, 0.7 μL of influenza A, and 1.3 μL of influenza B primer mixes were loaded per reaction chamber. Sodium polyacrylate granules (Macklin, China) were then placed in the waste chambers before sealing the chips. The chip halves were then aligned using an aluminum guide and hand-pressed together. They were further sealed using a 3000 kgf vertical press, with flat rubber O-rings placed on top of the chips during compression to distribute force from the platens to the edges of the chips.

### Imaging rig design and construction

The imaging rig was designed using various optical and electronic parts for fluorescence excitation and emission, and its construction is described in detail elsewhere [54]. Briefly, the optics are comprised of 465-nm LEDs, a 455/30-nm bandpass filter, 2-mm optical grade plastic light guides, a 520-nm long-pass filter, and a 10 MP USB camera mounted with an M12 wide-angle lens. These components were positioned with respect to one another using parts printed with polylactic acid (PLA) and acrylonitrile butadiene styrene (ABS) filament on a 3D-printer. The electronics are comprised of an Arduino microcontroller which controls the LEDs and the motor used for actuating the chip, and thermally regulates a custom-milled aluminum heat block.

### Viral RNA controls

Amplirun SARS-CoV-2 RNA controls (MBC137-R) were purchased from Vircell (Spain). Influenza A (No. 103001) and influenza B (No. 103003) RNA controls were purchased from Twist Biosciences (USA). All RNA controls were diluted into nuclease-free water (New England Biolabs, USA) according to the stock concentrations provided by the manufacturers (i.e., 13,000 copies per µL for SARS-CoV-2 RNA and 1,000,000 copies per µL for influenza A and influenza B).

For simulated saliva samples, approximately 1 mL of saliva was collected from healthy donors 30 min after oral rinsing with water. Clinical trial number: not applicable. Saliva aliquots were then spiked with 100 × inactivation solution and heated at 95 °C for 5 min. Inactivation solution was made by mixing 2.5 mL of 0.5 M TCEP-HCL, 1 mL of 0.5 M EDTA (pH 8.0), 1.15 mL of 5 N NaOH, and 0.35 mL of water [77]. RNA controls were then diluted along with inactivated saliva into RT-LAMP reactions.

### RT-LAMP assays

RT-LAMP reactions performed in a 384-well plate contained a 1 × master mix (10 mM ammonium sulfate, 10 mM potassium chloride, 8 mM magnesium sulfate, 0.1% Tween-20, 200 mM betaine monohydrate, pH 8.8), 5.6 mM dNTP mix (Invitrogen, USA), 1X SARS-CoV-2 LAMP primer mix, 0.3 units µL^−1^ RTx reverse transcriptase (M0380L, NEB, USA), and 0.32 units/µL Bst 2.0 DNA polymerase (M0537M, NEB, USA). Fifteen-microliter reaction volumes were incubated at 55 °C for 15 min and 65 °C for 35 min in a LightCycler 480 Instrument II Realtime PCR System (Roche Diagnostics GmbH). The wells of the plate were coated with optical pH sensor layers by drop-casting 10 μL of the precursor solution per well and heating at 70 °C for 10 min prior to the assays.

On-chip RT-LAMP reactions contained 1 × chip master mix (10 mM ammonium sulfate, 50 mM potassium chloride, 8 mM magnesium sulfate, 0.1% Tween-20, pH 8.8), 5.6 mM dNTP mix, 0.8 M betaine monohydrate, 0.3 units/µL RTx reverse transcriptase, and 0.64 U/μL Bst 2.0 polymerase. All chemicals except where otherwise indicated were purchased from Macklin, China. Varying numbers of RNA copies were used per reaction. Each chip was loaded with 105 μL of reaction mix and then rotated clockwise at 3400 RPM for 10 s, linearly ramped up to 11,500 RPM over 3 s, and then held at that angular frequency for 5 s. Microfluidic chips were actuated using a 3.3 V DC motor with an ABS 3D-printed rotor fixed to the shaft using cyanoacrylate adhesive. The motor’s speed was regulated by pulse-width modulated (PWM) voltage from an Arduino microcontroller and measured with a digital tachometer (20713A, Neiko, China).

After loading and actuating the centrifugal microfluidic chips with the reaction mixture, they were sealed with a die-cut circular piece of double-sided tape (93005LE, 3 M, USA). They were then inserted into the heat block of the imaging rig. A small pin was inserted between the chip’s edge and the heat block to ensure a secure fit. RT-LAMP experiments were initially incubated at 55 °C for 10 min followed by 65 °C for 50 min. A Python (v2.7.16) script was used to control data acquisition on the imaging rig. At 30-s intervals, commands were sent to the microcontroller to switch on the LEDs and to the camera module to photograph the chip. The LEDs were then turned off and the image was processed within the script. Seventy pixel-wide regions of interest (ROIs) were defined within the reaction chambers, and the background-subtracted average fluorescence of the ROIs was calculated. The values were tabulated for each image and exported to a CSV file.

For determining the limits of detection (LOD95) and 95% confidence intervals of each viral RNA titration, probit analysis in SPSS Statistics (version 29.0.1.0(171), IBM, USA) was employed.

### Data plotting and processing

RT-LAMP fluorescence curves were normalized with respect to the 12-min timepoint, by which time the optical pH sensors’ fluorescence had stabilized. These normalized curves were then plotted in GraphPad Prism (v9.4.1), which was also used for all other plotting and statistical analyses aside from the probit analysis. Additional fluorescence data processing was performed in Python (v3.10.4) to correct for discontinuities in relative fluorescence data due to bubble movement. Discontinuities were corrected as follows: first, each fluorescence data point was subtracted from the one before it, yielding stepwise difference data. Each of these stepwise differences (*d*_*i*_) was then compared to the preceding 8 data points according to the following formula:

$$\left|{d}_{i}-{D}_{\text{med}}\right|> 3*(\text{med}\{\left|{d}_{i-8}-{D}_{\text{med}}\right|,\cdots ,\left|{d}_{i-1}-{D}_{\text{med}}\right|\})$$

where ${D}_{\text{med}}=\text{med}\{{d}_{i-8},\cdots ,{d}_{i-1}\}$ and $\text{med}\{\}$ indicates the median of a set of points. If *d*_*i*_ satisfied the above inequality, then that stepwise difference was considered anomalous. Correlating these anomalies to discontinuities in the fluorescence data, the aberrant fluorescence values were then replaced with extrapolated values from the previous 10 data points using a linear regression. All subsequent data points were shifted by the same amount as the corrected value. This was performed for all fluorescence curves. Subsequently, the differential of the repaired fluorescence curves was calculated via the total variation regulation algorithm using a numeric differentiation library (PyNumDiff, v0.1.2.4) [78]. A threshold could then be applied to the resulting differential fluorescence curves to identify positive reactions in a quantitative manner.

## Results and discussion

### RT-LAMP readouts using optical pH sensors

**Fig. 2:**
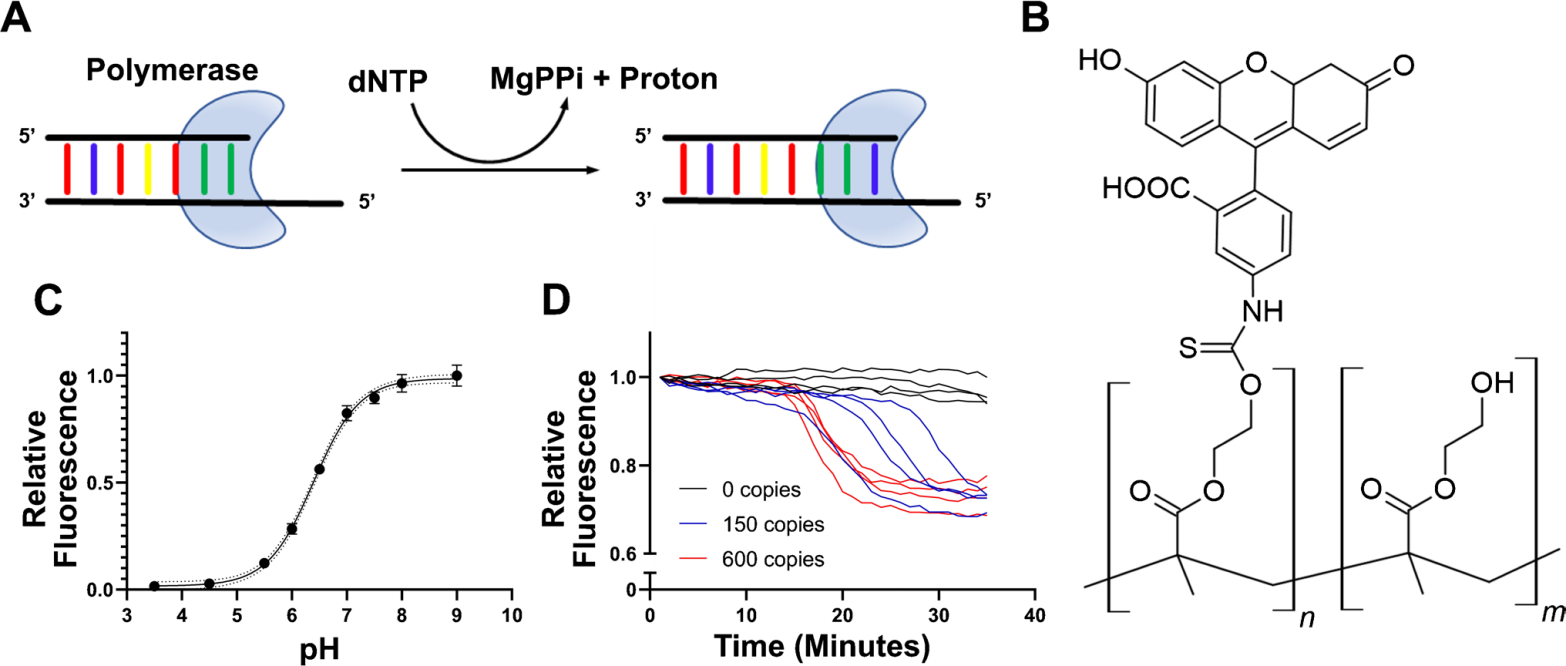
RT-LAMP readout generation via pH changes. **A** Schematic of dNTP incorporation illustrating the release of one magnesium pyrophosphate and one proton per nucleotide addition. **B** Chemical structure of FITC-pHEMA optical pH sensor. **C** Titration of FITC-pHEMA optical pH sensors drop-casted into polystyrene microwell plate. **D** Fluorescence measurements of RT-LAMP reactions containing indicated number of SARS-CoV-2 RNA copies and incubated in a 384-well plate with drop-casted optical pH sensors

LAMP reactions yield large quantities of amplified DNA products along with byproducts of nucleotide incorporation: magnesium pyrophosphate and hydrogen ions (Fig. 2a), [79]. In minimally buffered reactions, hydrogen ion release decreases pH which evokes fluorescence changes in optical pH sensors. By conjugating fluorescein isothiocyanate (FITC), a pH responsive fluorescent indicator, to poly(2-hydroxyethyl methacrylate), a hydrogel-forming molecule, and drop-casting into a microfluidic chamber, FITC-pHEMA (Fig. 2b) optical pH sensing layers were created. Performing a titration of the optical pH sensors confirmed that their fluorescent response spans the range over which pH changes occur in RT-LAMP (Fig. 2c), with a pK_a_ of 6.4 [52, 80]. The optical pH sensors were drop-casted into RT-LAMP reaction microwells and incubated with SARS-CoV-2 RNA controls. It was observed that RNA-loaded reactions showed a substantial decrease in fluorescence (Fig. 1d), confirming the utility of the optical pH sensors.

**Fig. 3:**
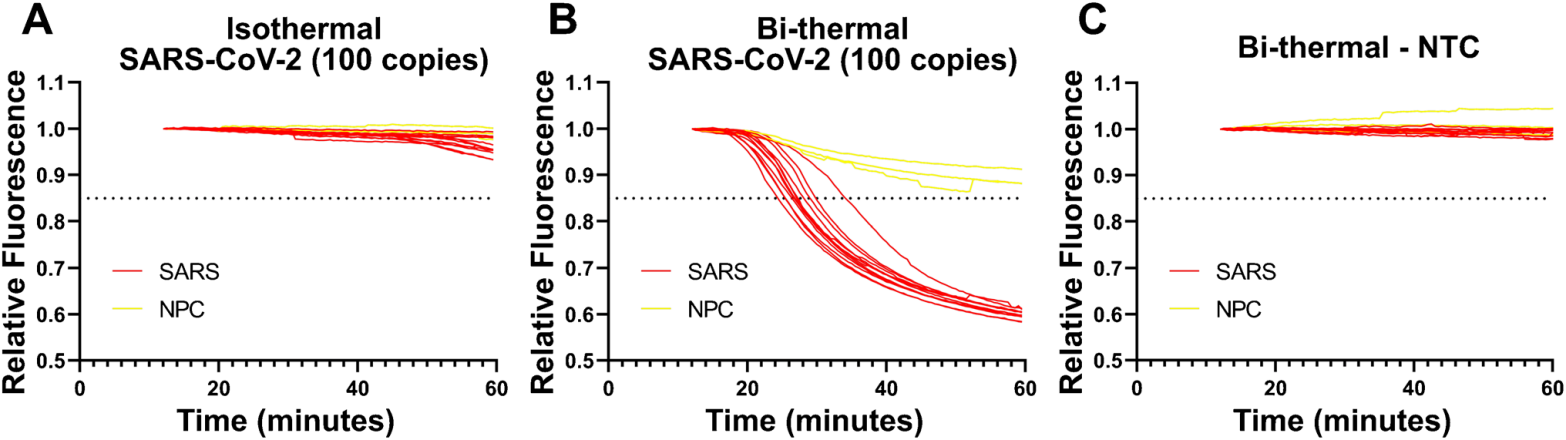
Comparison of isothermal and bi-thermal RT-LAMP reactions. Fluorescence curve plots for microfluidic chips incubated with 100 SARS-CoV-2 RNA copies per reaction chamber **A** isothermally and **B** bi-thermally, and **C** no template, bithermally

Also investigated were bithermal incubation strategies and their RT-LAMP performance. RT-LAMP reactions are typically performed isothermally, but we previously found that bi-thermal incubations enhanced their sensitivity [81]. Centrifugal microfluidic chips were made with SARS-CoV-2 primers in each reaction chamber, except for three no-primer control (NPC) reaction chambers without primers (one NPC chamber per microfluidic arm). These chips were then loaded with (i.e., 100 copies per reaction chamber) or without SARS-CoV-2 RNA and incubated. For the isothermal reaction, incubation at 65 °C for one hour was performed, whereas bi-thermal reactions were first incubated at 55 °C for 10 min followed by 65 °C for 50 min. Whereas the isothermal incubation did not yield robust amplification in any of the reaction chambers (Fig. 3a), the bi-thermal incubation encouraged substantial amplification within all the reaction chambers (Fig. 3b).

To verify that this enhanced amplification was not due to false positives, a bithermal incubation without template was also performed (Fig. 3c) which did not yield any amplification. Although isothermal amplification is a key advantage of LAMP, the inclusion of reverse transcriptase in RT-LAMP can hamper incubations at a single temperature which is optimized for Bst polymerase activity. Bithermal incubations are not substantially more technically complex to perform, since the setpoint of the incubator only needs to be programmatically adjusted. Given the improvements in sensitivity enabled by the bithermal incubation, this incubation scheme was used for all subsequent reactions.

### Titration of respiratory virus RNA

**Fig. 4:**
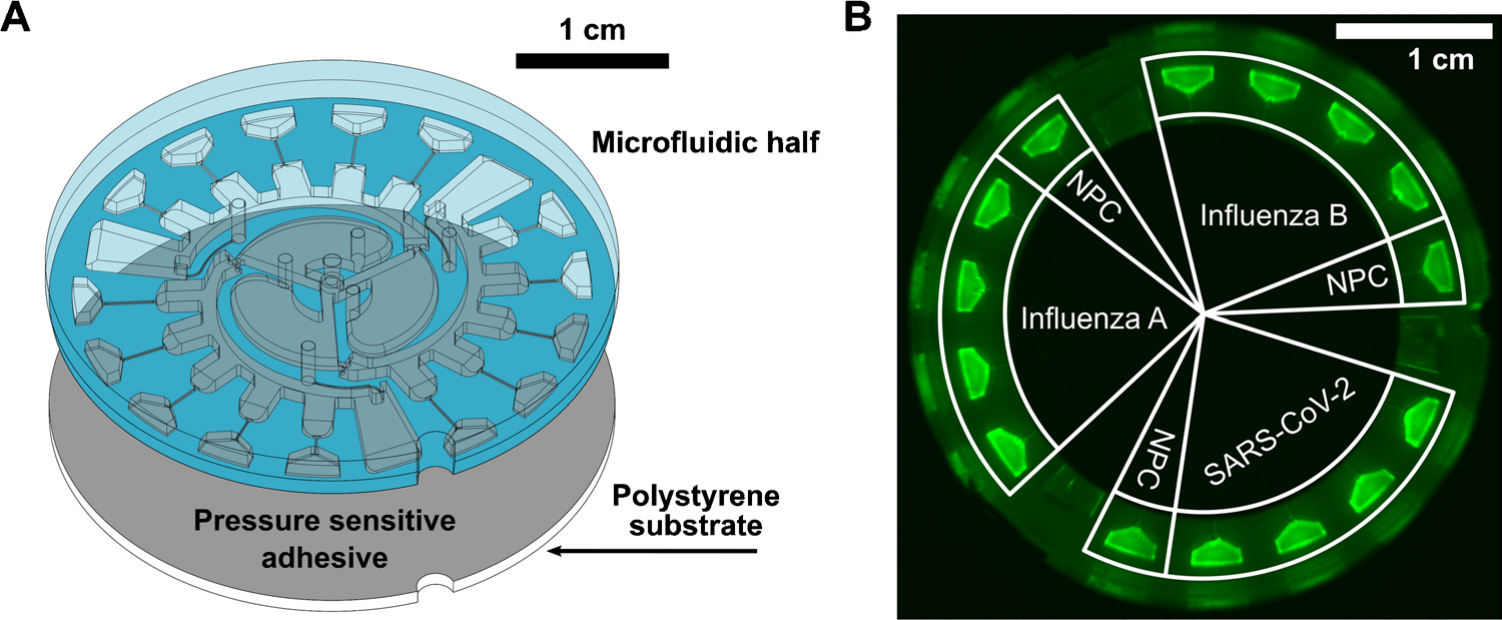
Microfluidic chip fabrication and functionalization. **A** Schematic depicting the assembly of the microfluidic chips and **B** the layout of primer sets withing the chip’s reaction chambers

For multiplexed detection of viral RNA, microfluidic chips were assembled by adhering an injection-molded microfluidic half to a polystyrene substrate using a pressure sensitive adhesive (Fig. 4a). The microfluidic halves were injection molded with general-purpose polystyrene. Polystyrene was used since it has favorable optical and mechanical properties and is biocompatible [82, 83]. Furthermore, it is not hygroscopic, unlike polymethylmethacrylate for example, which can trap moisture and must be thoroughly dried before injection molding; in this respect, injection molding with polystyrene is more facile [84, 85]. A polystyrene sheet was used for the base since it is easily fills into blanks for CNC milling. The general microfluidic chip design and actuation has been more thoroughly described in a previous work [54]. Briefly, the chip is 37.5 mm in diameter and features three identical branches with five reaction chambers per branch. After loading a liquid sample into the three central chambers which initiate each branch, the chip is spun at a first frequency, forcing the sample into finger-like metering chambers and excess fluid into waste chambers which terminate each branch. A second, higher frequency spin then causes the metered liquid to burst through capillary constrictions terminating each metering chamber, filling each of the separate reaction chambers. In this way, the sample can be consistently aliquoted into reaction chambers pre-loaded with differing reagents for multiplexed RT-LAMP assays. The chips are used only once since the pre-loaded reagents are expended after each assay and any RT-LAMP products could contaminate subsequent tests.

Primer sets were deposited into the separate reaction chambers of the microfluidic chips according to a specific layout (Fig. 4b) and allowed to dry before chip assembly. Each branch of the microfluidic chip targeted a separate viral RNA, with four replicates per virus. In addition, each branch contained one NPC chamber as a negative control. The primer sets were derived from other studies. The primer set for SARS-CoV-2 was obtained from Rabe and Cepko and targeted the ORF1a region [77]. The primer set for influenza A virus (IAV) was derived from one described by Jung et al., which targeted a conserved portion of the M gene [86]. The primer set for influenza B virus (IBV) was derived from a study by Ahn et al., which targeted a conserved portion of the NA gene [87]. The sequences for the influenza primers were adjusted slightly to match the strains used for the Twist RNA controls. The primer sets used for the RT-LAMP experiments are listed in supplementary Table 2. Viral RNA controls were titrated to determine a limit of detection for the multiplexed chip assays. Fluorescence curves were thus obtained for chips incubated with SARS-CoV-2 (Supp. Figure 1), IAV (Supp. Figure 2), and IBV (Supp. Figure 3).

Positive reactions could be discerned visibly by their sharp decreases in fluorescence; however, it was difficult to apply fixed fluorescence cutoffs since the curves tended to differentially drift over time. To enable reliable determination of positive reactions, the fluorescence data was smoothed and differentiated using the total variation regulation (TVR) algorithm [78, 88]. Differentiation mitigated the cumulative effects of fluorescence drifts, and the fluorescence derivative plots maintained the qualitative outcomes of the original fluorescence plots. The fluorescence curves which visibly indicated a positive LAMP reaction by steep drops in fluorescence yielded differential curves which had sustained stretches of negative values.

**Fig. 5:**
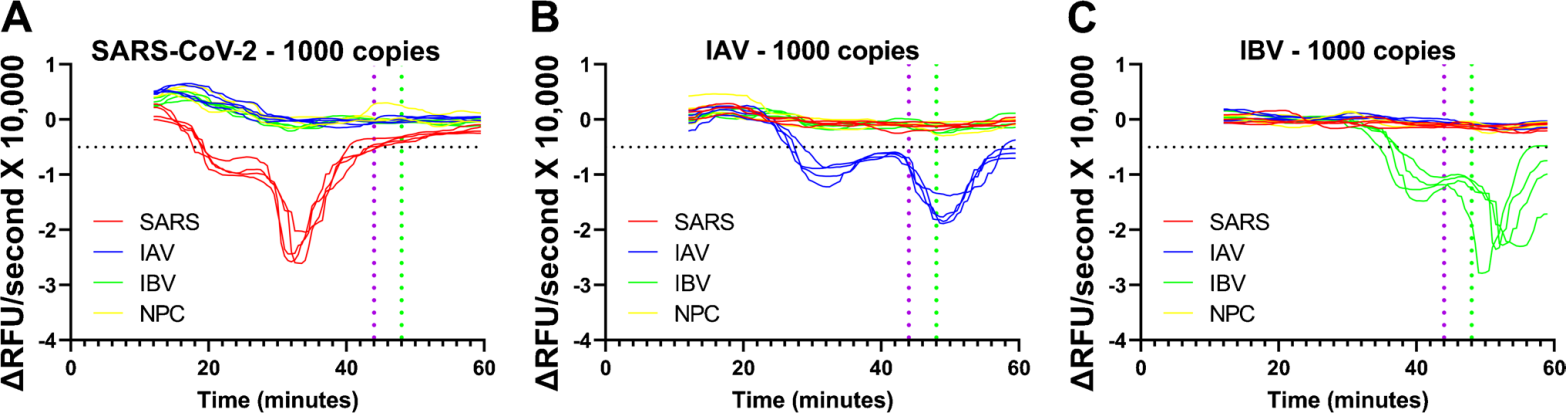
TVR numerical differentiation of fluorescence curves for viral RNA RT-LAMP reactions. Numerical derivative plots of fluorescence curves for reactions containing 1000 copies of **A** SARS-CoV-2, **B** IAV, and **C** IBV RNA. Horizontal dotted line indicates threshold for positivity; vertical dotted lines indicate threshold for false positivity of SARS-CoV-2 and IAV reactions (purple) and IBV reactions (green)

To quantitate reactions, a horizontal threshold was set at − 0.5 × 10^−4^ ΔRFU/second and to eliminate false positives, a temporal threshold was set at 44 min for SARS-CoV-2 (Fig. 5a, Supp. Figure 7). The same thresholds were then applied to IAV (Fig. 5b, Supp. Figure 8) and IBV reactions (Fig. 5c, Supp. Figure 9), with the temporal threshold being increased to 48 min for IBV reactions since they were generally somewhat slower. Beyond the temporal thresholds, any positive reactions were not considered true positives. These temporal thresholds compensated for spurious amplifications that occurred in some reactions (see Supp. Figures 8d, 9b), thereby improving accuracy. Using these thresholds, titration curves were generated, and 95% limits of detection (LOD95s) were determined with a probit analysis.

**Fig. 6:**
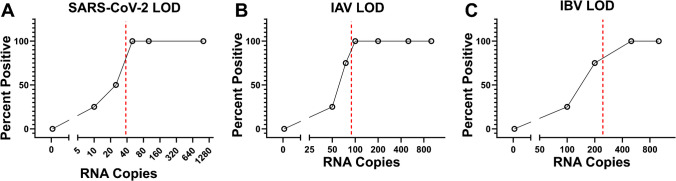
Titration curves for **A** SARS-CoV-2, **B** IAV, and **C** IBV. Red dotted lines indicate the limit of detection (LOD95) for each viral RNA

The LOD95 was 38 copies/reaction (CI 95%: 27–85 copies/reaction) for SARS-CoV-2 (Fig. 6a), 89 copies/reaction (CI 95%: 72–486 copies/reaction) for IAV (Fig. 6b), and 245 copies/reaction (CI 95%: 182–486 copies/reaction) for IBV (Fig. 6c). Assuming a purification strategy which enables fivefold concentration of viral nucleic acids and loading of 5 µL of concentrate per reaction chamber, then detectable viral loads of 1.5 × 10^3^, 3.6 × 10^3^, and 9.8 × 10^3^ copies × mL^−1^ could be achieved for SARS-CoV-2, IAV, and IBV, respectively. This is within the lower range of viral titers observed for these viruses [89] and indicates that the multiplex assay has potential clinical utility.

Furthermore, these sensitivities are comparable to those obtained by other groups which had previously used microfluidics for multiplexed POC diagnostics of respiratory pathogens (Table 1). However, whereas these studies relied on chips fabricated with more expensive techniques or using invasive and expensive probes, we have utilized a more scalable and economical desktop injection–molding technique and applied readily available, low-cost, and non-invasive pH sensors integrated into the microfluidic assembly. These results therefore validate the technique and confirm the compatibility of fluorescent optical pH sensors with RT-LAMP.

**Table 1:** Comparison of representative microfluidic POC platforms for multiplexed respiratory pathogen diagnostics, published since the emergence of SARS-CoV-2

Number of pathogens multiplexed	Detection chemistry	Limit of detection (copies/μL of sample)	Specificity	Assay duration	Readout mechanism	Microfluidic type	Fabrication method	Refs
2	RPA + LAMP	10 (measles virus)	96%—SARS-CoV-2; 100%—measles virus	60 min	Fluorescence (nucleic acid dye)	Centrifugal microfluidic chip	CNC milling	[90]
3	RT-LAMP	50	100%	60 min	Colorimetry (pH-sensitive dye)	Paper microfluidic	CNC drawing machine	[91]
2	RT-LAMP	10	100%	30 min	Fluorescence (nucleic acid dye)	Pneumatic cartridge	3D printing	[92]
4	RT-LAMP	100 (SARS-CoV-2)	100%	90 min	Fluorescence (nucleic acid dye)	Centrifugal microfluidic chip	CNC milling	[93]
3	RT-LAMP	100 (SARS-CoV-2), 100 pfu ml^−1^ (IAV, IBV)	100%	70 min	Fluorescence (nucleic acid dye)	Pneumatic cartridge	CNC milling	[94]
7	RT-LAMP	10 pfu ml^−1^ (IAV/H3N2)	95%—SARS-CoV-2; 100%—IAV	80 min	Fluorescence (nucleic acid dye)	Centrifugal microfluidic chip	CNC milling	[95]
3	RT-PCR	2 (SARS-CoV-2, IAV), 24 (IBV)	95%—SARS-CoV-2; 100%—IAV; 98%—IBV	30 min	Fluorescence (hydrolysis probes)	Pneumatic cartridge	Thermoforming	[96]
7	RT-LAMP	10	100%	40 min	Fluorescence (nucleic acid dye)	Centrifugal microfluidic chip	N/A (Commercially sourced)	[97]
3	RT-LAMP	2000*	100%	30 min	Colorimetry (complexometric indicator)	Pneumatic cartridge	Soft lithography	[98]
21	RT-PCR	1	100%	90 min	Fluorescence (hydrolysis probes)	Mechanically actuated cartridge	Injection-molded (commercially)	[99]
3	RT-LAMP	7.6 (SARS-CoV-2), 17.8 (influenza A), 49 (influenza B)	100%	60 min	Fluorescence (optical pH sensor)	Centrifugal microfluidic chip	Injection-molded (desktop machine)	This work

*Only a single RNA concentration was tested for each virus. *IAV*, influenza A virus; *IBV*, influenza B virus

### Compatibility of RT-LAMP reactions with inactivated saliva

To test the practicality of the on-chip viral RNA detection methodology, the compatibility of the RT-LAMP reactions with inactivated saliva samples was determined. Saliva is easy to collect and useful for viral diagnostics, making it suitable for POC diagnostics [100]. However, in POC settings, it is ideal to avoid extraction procedures, since this can make tests more complicated and less scalable [101]. In another work, saliva samples were directly used in RT-LAMP reactions by first mixing with a solution of TCEP and EDTA, and incubating at 95 °C [77]. To test if this saliva inactivation procedure would work for reactions performed on the chips, saliva samples from three donors were collected 30 min after an oral rinse with water. These samples were then aliquoted into tubes, mixed with inactivation solution, and heat-treated. These treated samples were loaded into reactions along with quantities of each viral RNA that had previously yielded 100% sensitivity.

**Fig. 7:**
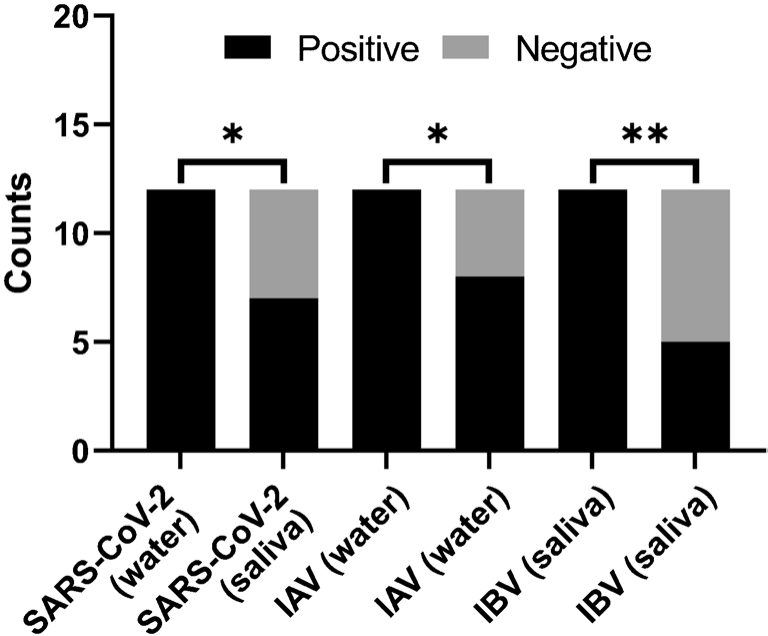
Comparison of assay sensitivity when using RNA controls without (water) and with (saliva) inactivated saliva. Performed using Fisher’s exact test, one-sided. *p value < 0.05, **p value < 0.01

For each saliva sample, three plots were generated, one for each viral RNA. Both normalized fluorescence curves (Supp. Figure 10) and the numerical derivatives of these curves (Supp. Figure 11) were plotted to thoroughly assess outcomes. In contrast to the results obtained in the study where this methodology was tested, it was found that inactivated saliva was not always compatible with the RT-LAMP reactions [77]. Although some of the samples yielded results that were reasonably consistent with the reactions without saliva (Supp. Figure 11a), other samples completely inhibited the reactions within the incubation timeframe (Supp. Figure 11c). Comparing the saliva reactions against control reactions which did not use saliva (Supp. Figure 12), a statistically significant decrease in sensitivity was observed for all viral RNAs (Fig. 7).

In real-world settings, this would entail false negatives, and it can be concluded that inactivated saliva samples are not ideal for RT-LAMP reactions. Samples should be made consistent before loading into chips and extraction procedures should be incorporated into POC technologies. Nucleic acid isolation is therefore an important consideration since the benefits are twofold: it ensures samples do not inhibit reactions and can further improve sensitivity by concentrating low viral loads. Technologies have been developed for POC nucleic acid purification, and it would be worthwhile to adapt these strategies in the future [102, 103].

## Conclusions

In this work, multiplexed detection of viral RNAs via RT-LAMP was achieved using optical pH sensors integrated with centrifugal microfluidic chips. The physical separation of primer sets into different reaction chambers enabled the discrimination of influenzas A and B and SARS-CoV-2 RNAs. RT-LAMP reactions elicited decreases in optical pH sensor fluorescence allowing reaction outcomes to be determined by setting thresholds for the numerical derivatives of the real-time fluorescence curves. However, using saliva samples the results were mixed and it could be concluded that saliva inactivation does not guarantee its compatibility with RT-LAMP reactions. The limits of detection obtained for the primer sets, in principle, enable clinical applications and testing of clinical samples will be performed in the future to evaluate the utility of these microfluidic chips.

A key hindrance to the translation of promising diagnostic technologies, especially those which test for constantly evolving respiratory pathogens, is a lack of methods which bridge the gap between research and commercialization. The demonstration of highly scalable desktop injection-molded chips for this application reveals a methodology that can be applied for future health crises. The integration of fluorescent optical pH sensors expands the repertoire of readouts that future studies can leverage, and we anticipate that this work will benefit the ongoing battle against emerging infectious diseases.

## Supplementary Information

Below is the link to the electronic supplementary material.

Supplementary file1 (DOCX 18791 KB)

## Data Availability

No datasets were generated or analysed during the current study.
