# Supplementary material for: Multiplexed detection of respiratory virus RNA using optical pH sensors and injection-molded centrifugal microfluidics
Source: Mikrochim Acta. 2025 Feb 12;192(3):151. doi: 10.1007/s00604-025-06996-3 (PMC11821746; doi:10.1007/s00604-025-06996-3)
Supplement: Supplementary file 1 — Supplementary file1 (DOCX 18791 KB) [file 604_2025_6996_MOESM1_ESM.docx]

**Supplementary Information**

**Multiplexed Detection of Respiratory Viruses Using Optical pH Sensors and Injection Molded Centrifugal Microfluidics**

Gianmarco D. Suarez,^a^ Yuki Yu Kiu Tang,^b^ Steevanson Bayer,^a^ Peter Pak-Hang Cheung^c^ and Stefan Nagl^a^*

^a^Department of Chemistry, The Hong Kong University of Science and Technology, Clear Water Bay, Kowloon, Hong Kong

^b^Quommni Technologies Limited, Tsuen Wan, New Territories, Hong Kong

^c^Department of Chemical Pathology, The Chinese University of Hong Kong, Shatin, New Territories, Hong Kong

*To whom correspondence may be addressed. Email: chnagl@ust.hk

**Table of Contents**

[**Normalized Fluorescence Curves for Viral RNA Titration** 3](#_Toc187806817)

[**Supplementary Figure 1: Titration of SARS-CoV-2 RNA copies in on-chip RT-LAMP reactions** 4](#_Toc187806818)

[**Supplementary Figure 2: Titration of influenza A RNA copies in on-chip RT-LAMP reactions** 5](#_Toc187806819)

[**Supplementary Figure 3: Titration of influenza B RNA copies in on-chip RT-LAMP reactions** 6](#_Toc187806820)

[**Data Processing for RT-LAMP Reactions** 6](#_Toc187806821)

[**Supplementary Figure 4: Interference by fluoresence data discontinuities with numerical derivative analysis** 7](#_Toc187806822)

[**Supplementary Figure 5: Fluorescence data discontinuities correspond to bubble movements and changes** 8](#_Toc187806823)

[**Supplementary Figure 6: Removal of anomalous spikes in numerical derivative curves** 9](#_Toc187806824)

[**Supplementary Figure 7: TVR numerical differentiation of fluorescence curves for assessing SARS-CoV-2 titration outcomes.** 10](#_Toc187806825)

[**Supplementary Figure 8: TVR numerical differentiation of fluorescence curves for assessing influenza A titration outcomes** 11](#_Toc187806826)

[**Supplementary Figure 9: TVR numerical differentiation of fluorescence curves for assessing influenza B titration outcomes** 12](#_Toc187806827)

[**Data for Inactivated Saliva Compatibility Experiments** 13](#_Toc187806828)

[**Supplementary Figure 10: Inactivated saliva RT-LAMP compatibility fluorescence curves** 13](#_Toc187806829)

[**Supplementary Figure 11: Numerical derivative analysis to assess inactivated saliva compatibility with RT-LAMP** 14](#_Toc187806830)

[**Supplementary Figure 12: Numerical derivative analysis of RT-LAMP replicates** 15](#_Toc187806831)

[**Supplementary Table 1: Cost assessment of services, equipment, materials and reagents for injection molded centrifugal microfluidic chips** 16](#_Toc187806832)

[**Supplementary Table 2: Sequences of primers used in this study for RT-LAMP** 17](#_Toc187806833)

**Normalized Fluorescence Curves for Viral RNA Titration**

To determine the sensitivity of the RT-LAMP reactions within the microfluidic chips, titrations were performed for the influenza A (IAV), influenza B (IBV), and SARS-CoV-2 RNA controls. In the resulting normalized fluorescence curves (Supplementary Figures 1, 2, 3), positive reactions could be visibly discerned by their characteristic precipitous drop in fluorescence. However, it was difficult to establish a numerical cutoff for positivity without compromising either sensitivity or specificity. This was due to a tendency for reactions’ fluorescence to drift over time, even in NPC reactions. This drift could be attributed to minute shifts of the chip due to thermal expansion during incubation. Therefore, to enable the systematic determination of positive reactions, we sought to nullify these drifts through data processing, as described below.


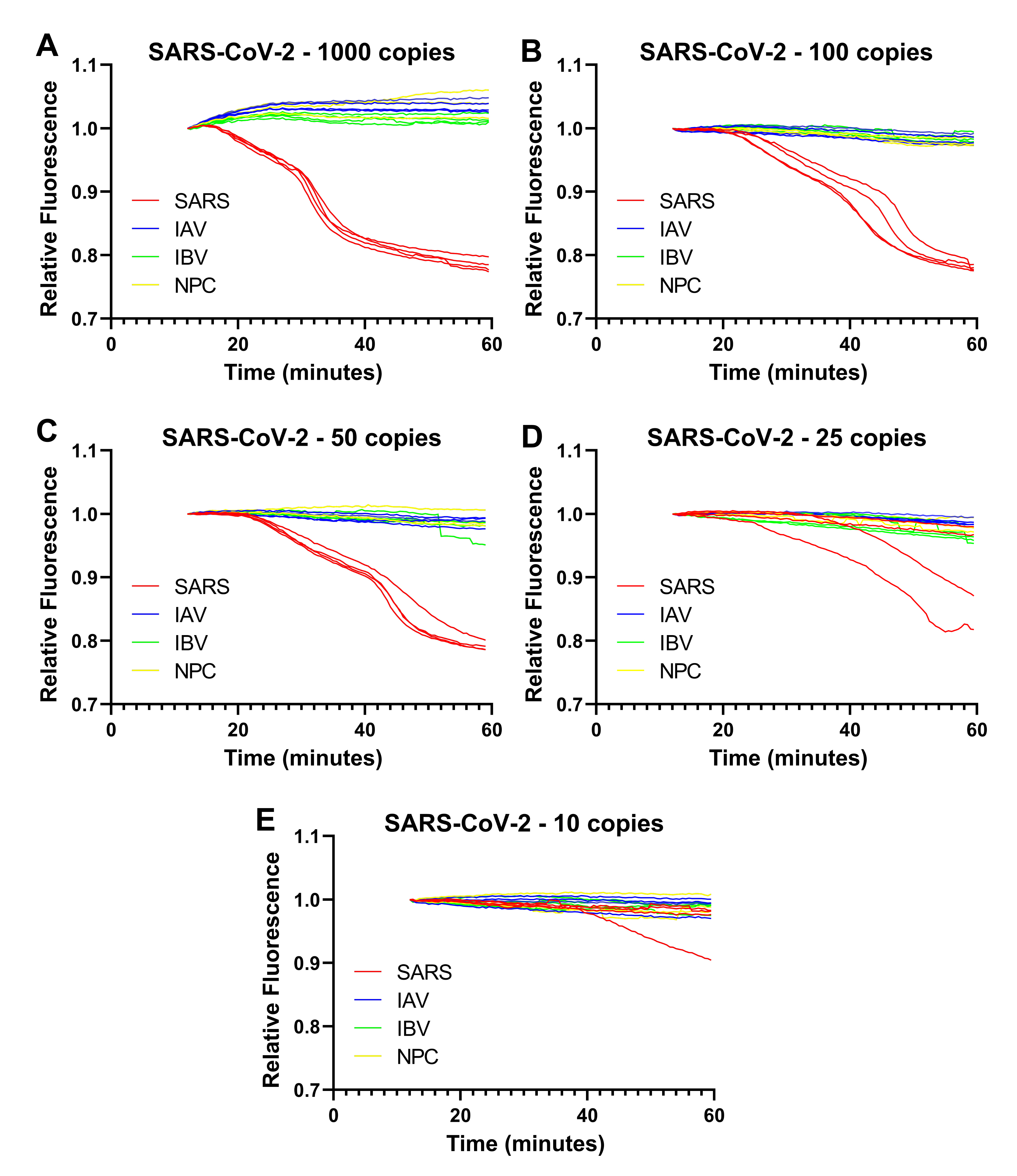


**Supplementary Figure 1:** Titration of SARS-CoV-2 RNA copies in on-chip RT-LAMP reactions. Relative fluorescence curve plots for reactions containing (a) 1000, (b) 100, (c) 50, (d) 25 and (e) 10 RNA copies.


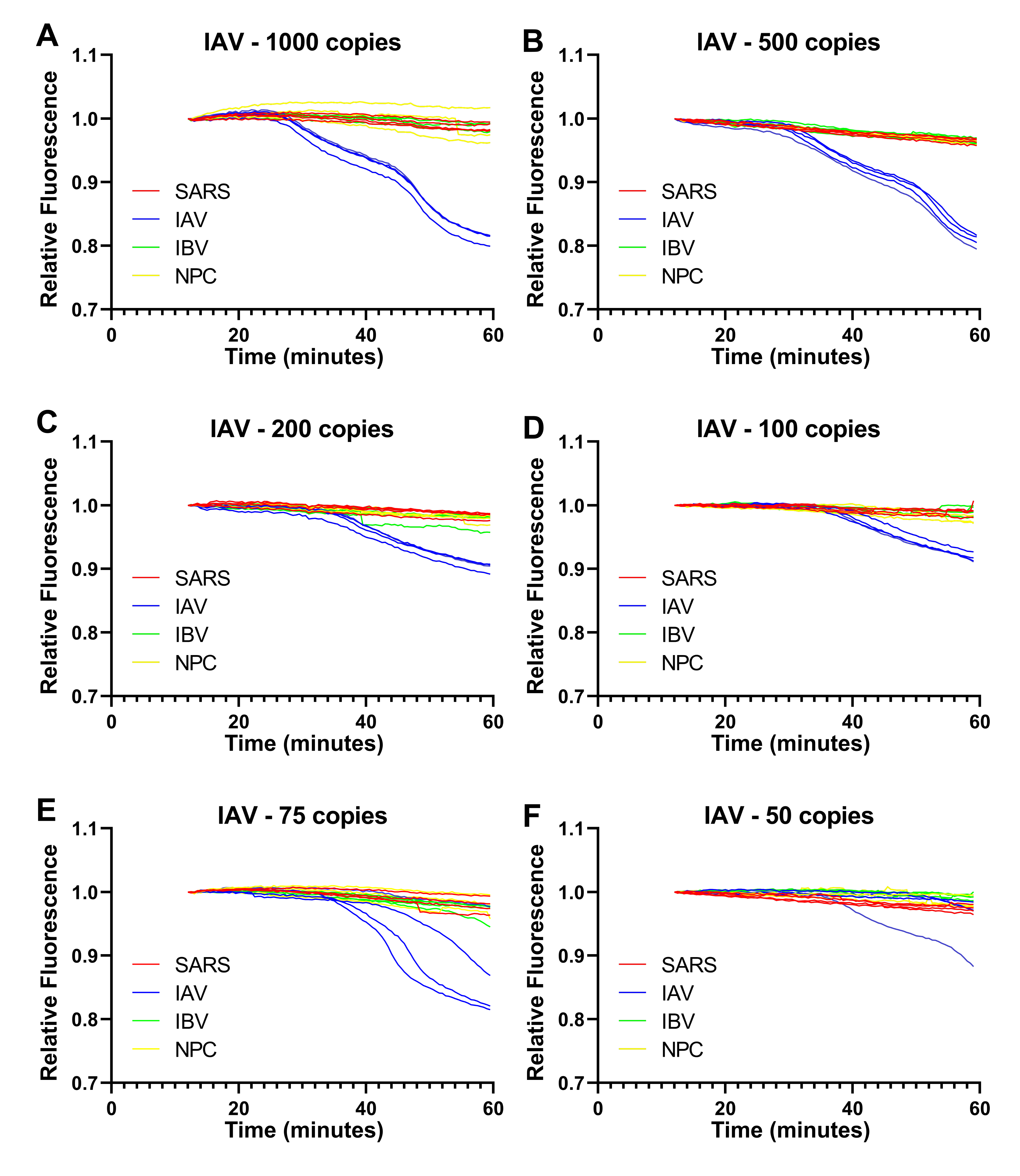


**Supplementary Figure 2:** Titration of influenza A RNA copies in on-chip RT-LAMP reactions. Relative fluorescence curve plots for reactions containing (a) 1000, (b) 500, (c) 200, (d) 100, (e) 75 and (f) 50 RNA copies.


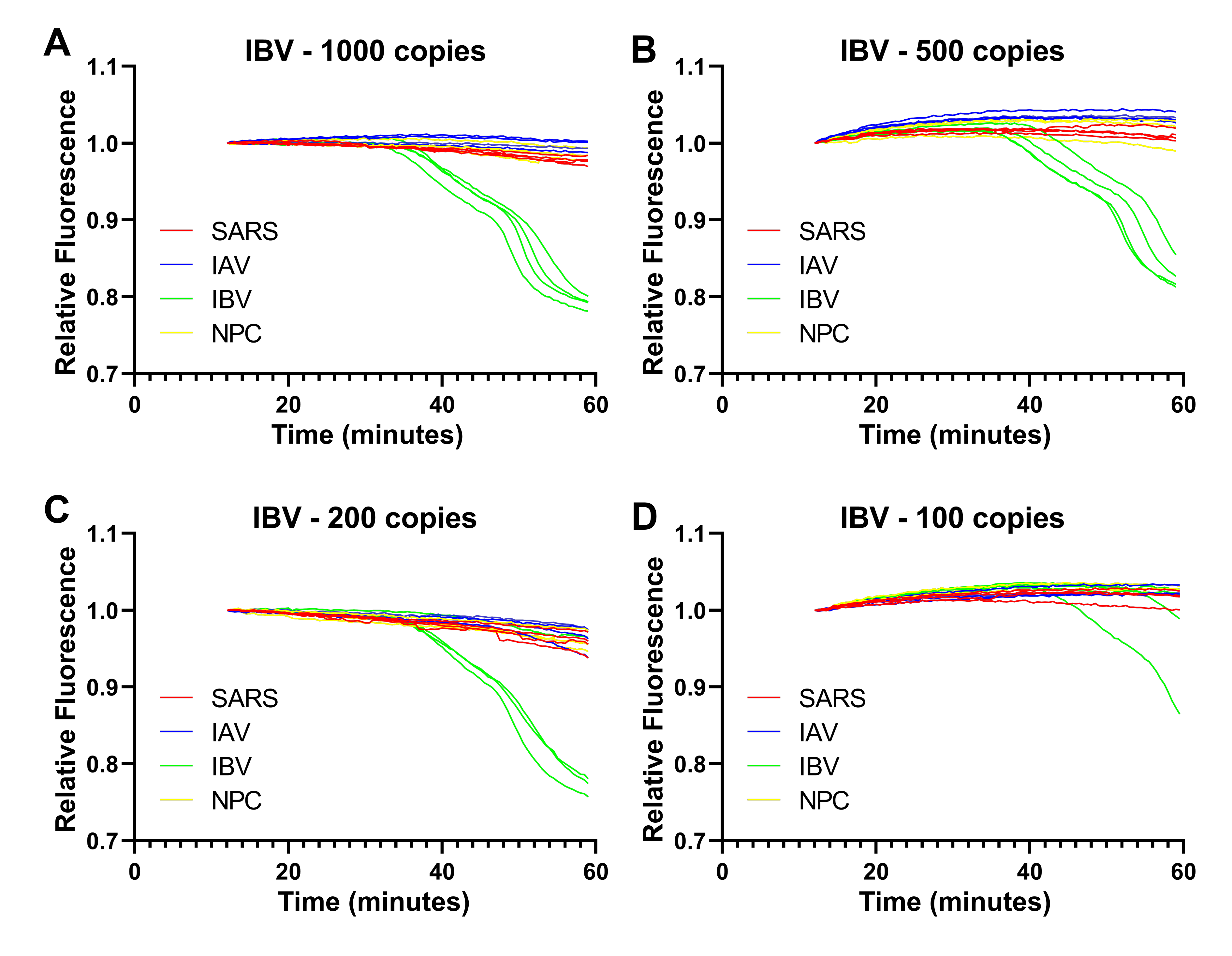


**Supplementary Figure 3:** Titration of influenza B RNA copies in on-chip RT-LAMP reactions. Relative fluorescence curve plots for reactions containing (a) 1000, (b) 500, (c) 200 and (d) 100 RNA copies.

**Data Processing for RT-LAMP Reactions**

Given the gradual drifts in fluorescence over time, we reasoned they could be mitigated by taking the first derivative of the fluorescence curves. In various other assays, numerical derivatives are used to extract useful information for diagnostic purposes.[1–5] Taking the first derivative could cancel the cumulative effects of these drifts from the data while accentuating the characteristic drops in fluorescence for positive reactions. For numerical differentiation, a Python script using the PyNumDiff library was implemented.[6] The total variation regulation (TVR) algorithm was used to smooth and differentiate the data, because of its ability to suppress noise without compromising the fidelity of the data.[7]

The overall behavior of the original curves (Supp. Fig. 4a) was retained in the smoothed curves (Supp. Fig. 4b); however, it was found that some fluorescence curves generated anomalous peaks in their derivatives (Supp. Fig. 4c). The peaks corresponded to stepwise discontinuities in the fluorescence curves which persisted through smoothing and ultimately into the numerical derivative curve. These peaks hindered the application of a threshold for positivity since they registered otherwise negative reactions as positive.

The cause of the discontinuities was determined. Using an IBV and NPC curve from the IAV 200-copy experiment for reference, prominent discontinuities were identified in the data (Supp. Fig. 5a) and visualized as anomalous peaks in the stepwise difference curve, obtained by subtracting each fluorescence value from the previous one (Supp. Fig. 5b). Cross-referencing the images from these particular assays, it was found that at the timepoints corresponding to the discontinuities, bubbles within the chambers appeared, moved, or changed in size (Supp. Fig. 5c, d). Likewise, for all other assays and fluorescence curves, stepwise discontinuities in the data could be tied to bubbles in reaction chambers. The formation of bubbles within LAMP reaction chambers has been observed in other studies.[8, 9] Given that the bubbles disrupted the curves, their effects were mitigated by implementing a Python script to process the fluorescence curve data.

To do this, anomalous peaks in stepwise difference data were identified by comparing them to their immediate neighbors. If a stepwise difference deviated sufficiently from median of the neighbors (by more than 3-fold the median of the differences between the neighbors and their median), then the corresponding discontinuity in the fluorescence data was identified. The data point at the discontinuity was then set to a linearly extrapolated value based on the previous data points and all subsequent data points were shifted by the same amount as the corrected data point. This yielded corrected curves (Supp. Fig. 5e) which did not produce anomalous peaks (Supp. Fig. 5f).


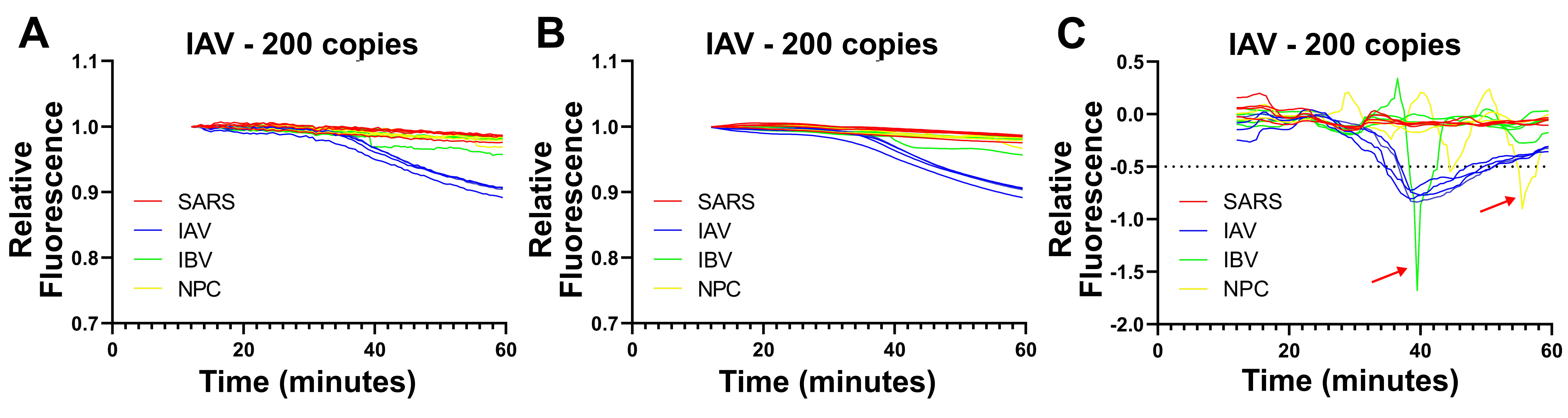


**Supplementary Figure 4:** Interference by fluoresence data discontinuities with numerical derivative analysis. Fluorescence curve plots for (a) normalized fluorescence data and (b) normalized and TVR smoothed fluorescence data, and (c) plot of numerical derivative of smoothed fluorescence data. Arrows indicate prominent peaks in numerical data corresponding to discontinuities in fluorescence data. The horizontal dotted line indicates the threshold for positivity.


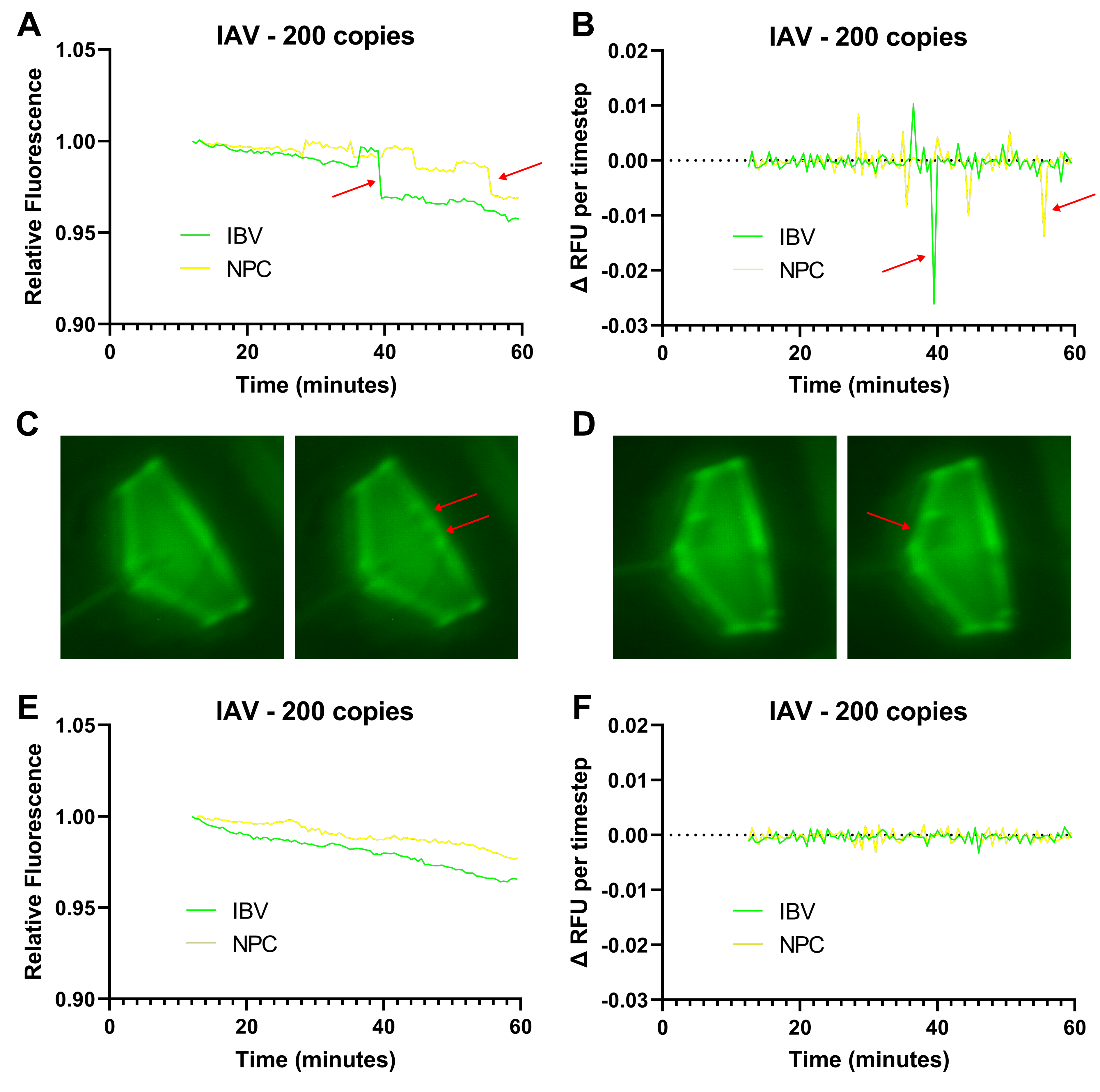


**Supplementary Figure 5:** Fluorescence data discontinuities correspond to bubble movements and changes. (a) Plot of relative fluorescence curves with discernible discontinuities and (b) the corresponding stepwise difference plot. Arrows indicate spikes in the difference plot which correspond to discontinuties in the fluorescence curves. Sequential images of reaction chambers corresponding to the (c) IBV and (d) NPC fluorescence curves. Arrows indicate bubbles which have appeared, moved, or changed in size. (e) Plot of relative fluorescence curves after correcting for discontinuties due to bubbles and (f) the corresponding stepwise difference plot.

When processed using the TVR algorithm, the assay data yielded smoothed curves which behaved consistently with the original fluorescence curves (Supp. Fig. 6a). Additionally, the anomalous peaks in the numerical derivative curves were eliminated, while the true positive IAV reaction curves retained their positivity (Supp. Fig. 6b).


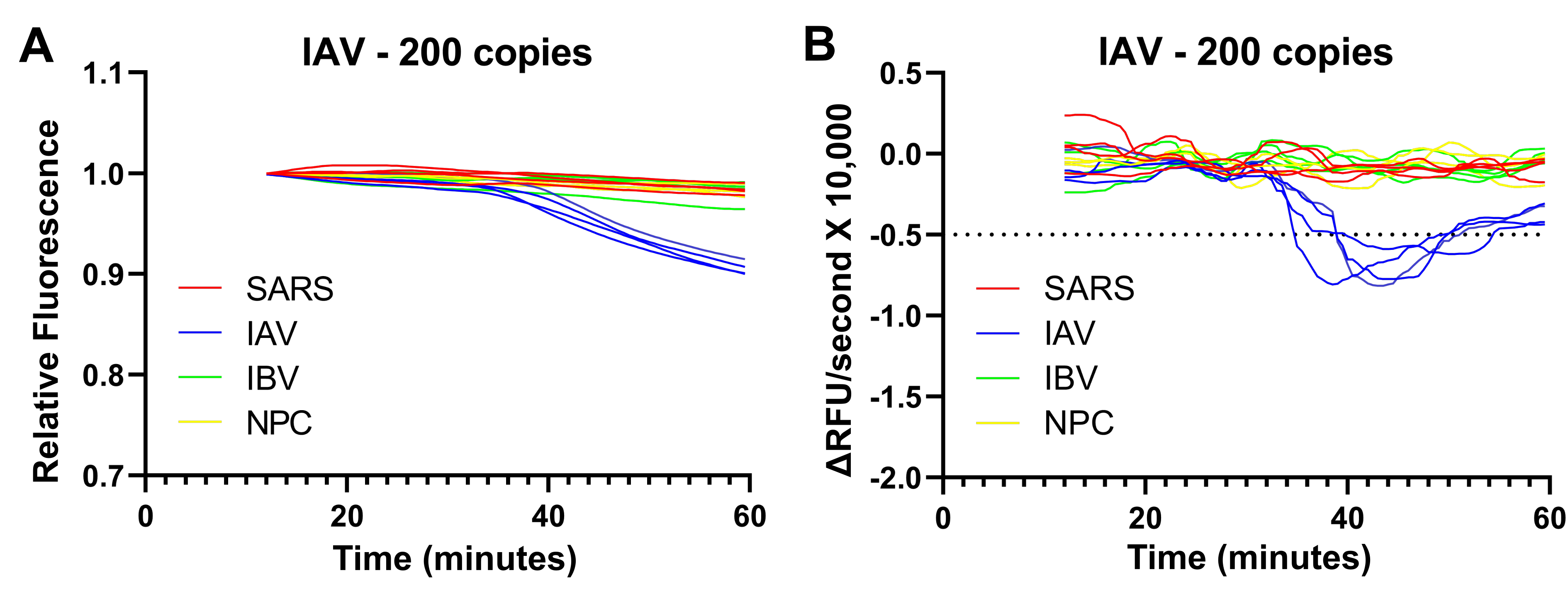


**Supplementary Figure 6:** Removal of anomalous spikes in numerical derivative curves. (a) Plot of repaired fluorescence curve data and (b) the corresponding numerical derivatives. Horizontal dotted line indicates the threshold for positivity.

The original fluorescence data for the RNA titrations was then processed into numerical derivative curves (Supp. Figs. 7, 8, 9) on which thresholds for positivity and false positivity could be set.


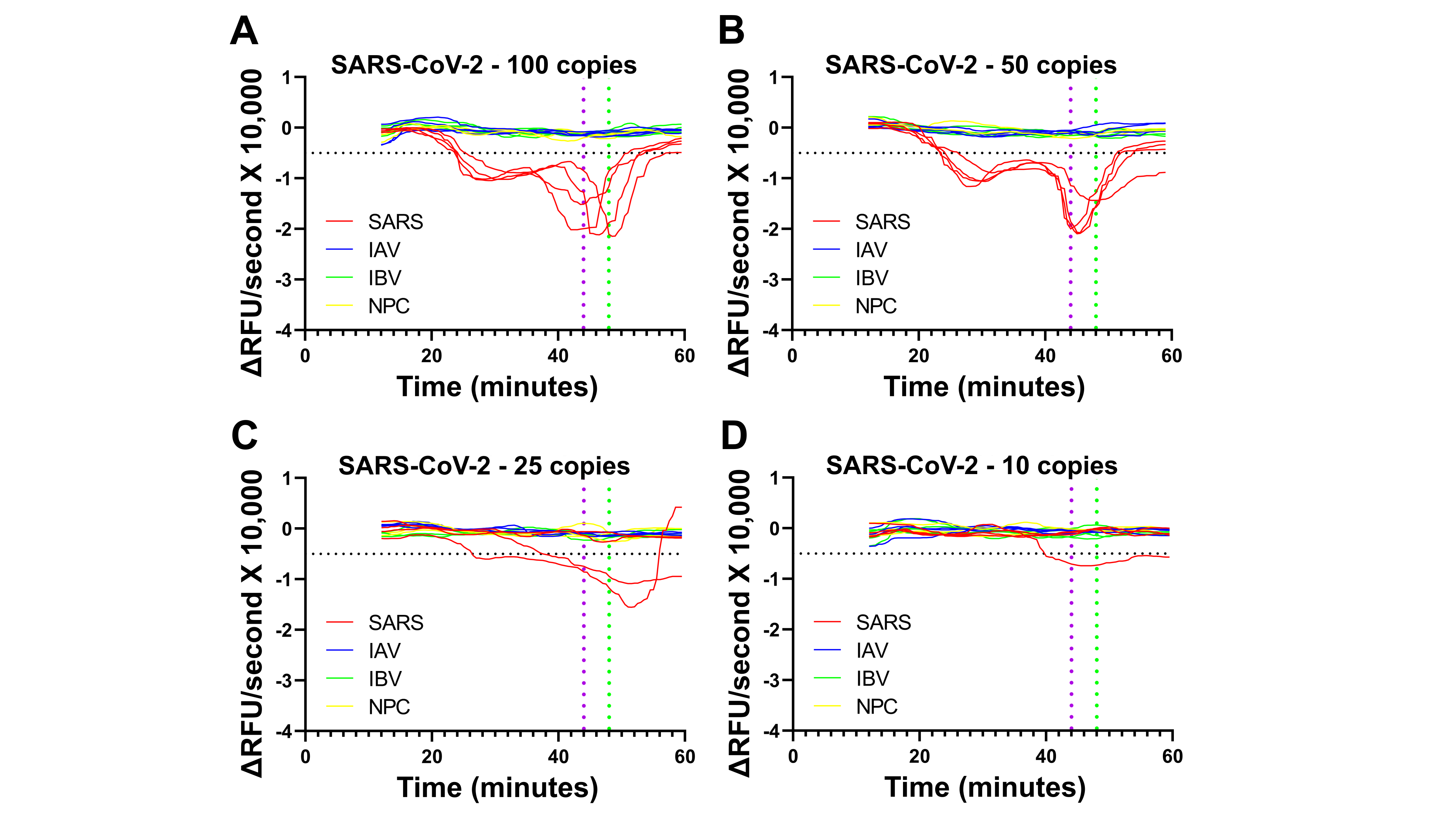


**Supplementary Figure 7:** TVR numerical differentiation of fluorescence curves for assessing SARS-CoV-2 titration outcomes. Numerical derivative plots of fluorescence curves for reactions containing (a) 100, (b) 50, (c) 25, and (d) 10 copies of RNA. Horizontal dotted line indicates threshold for positivity; vertical dotted lines indicate threshold for false positivity of SARS-CoV-2 and IAV reactions (purple) and IBV reactions (green).


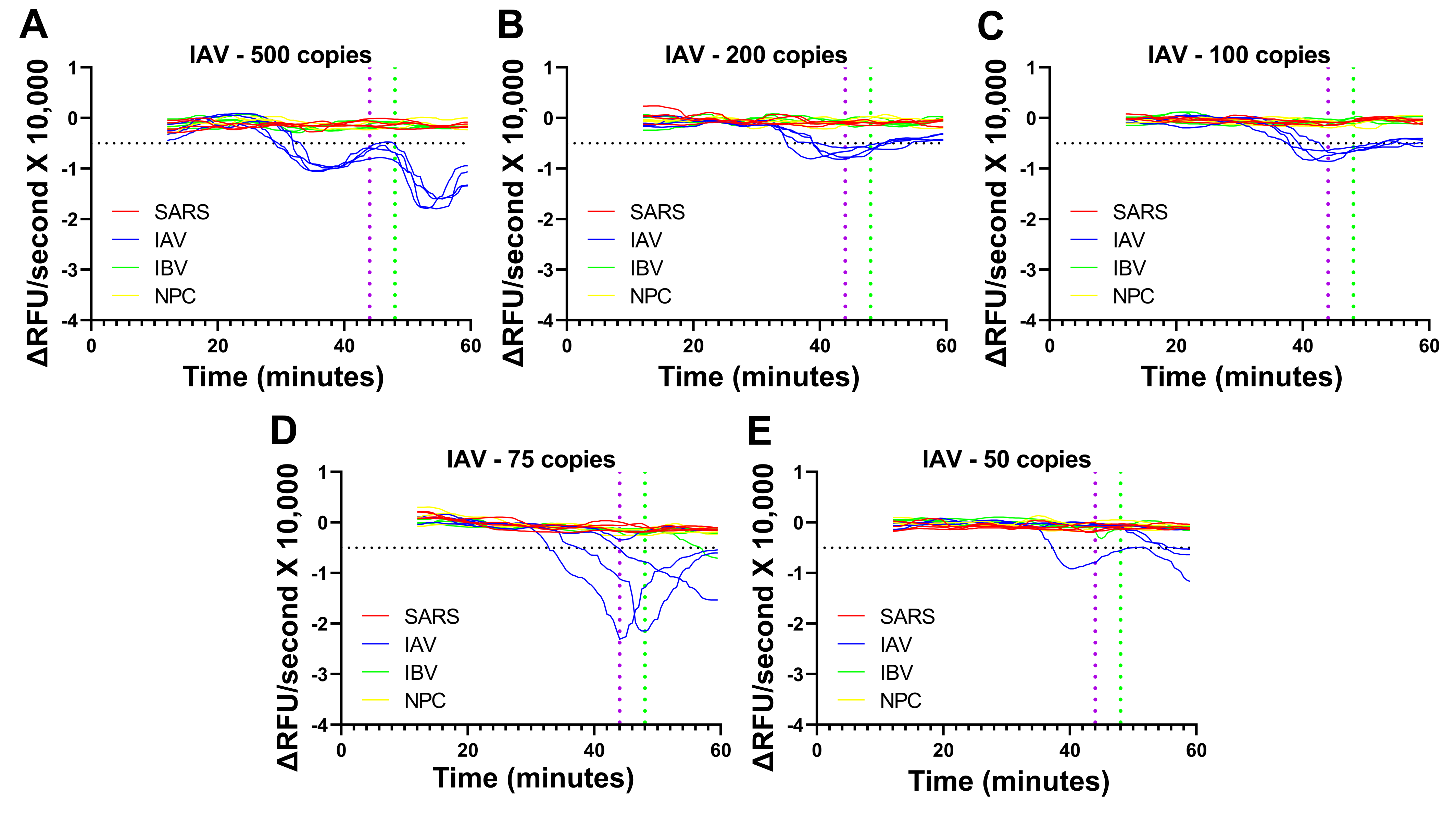


**Supplementary Figure 8:** TVR numerical differentiation of fluorescence curves for assessing influenza A titration outcomes. Numerical derivative plots of fluorescence curves for reactions containing (a) 500, (b) 200, (c) 100, (d) 75, and (e) 50 copies of RNA. Horizontal dotted line indicates threshold for positivity; vertical dotted lines indicate threshold for false positivity of SARS-CoV-2 and IAV reactions (purple) and IBV reactions (green).


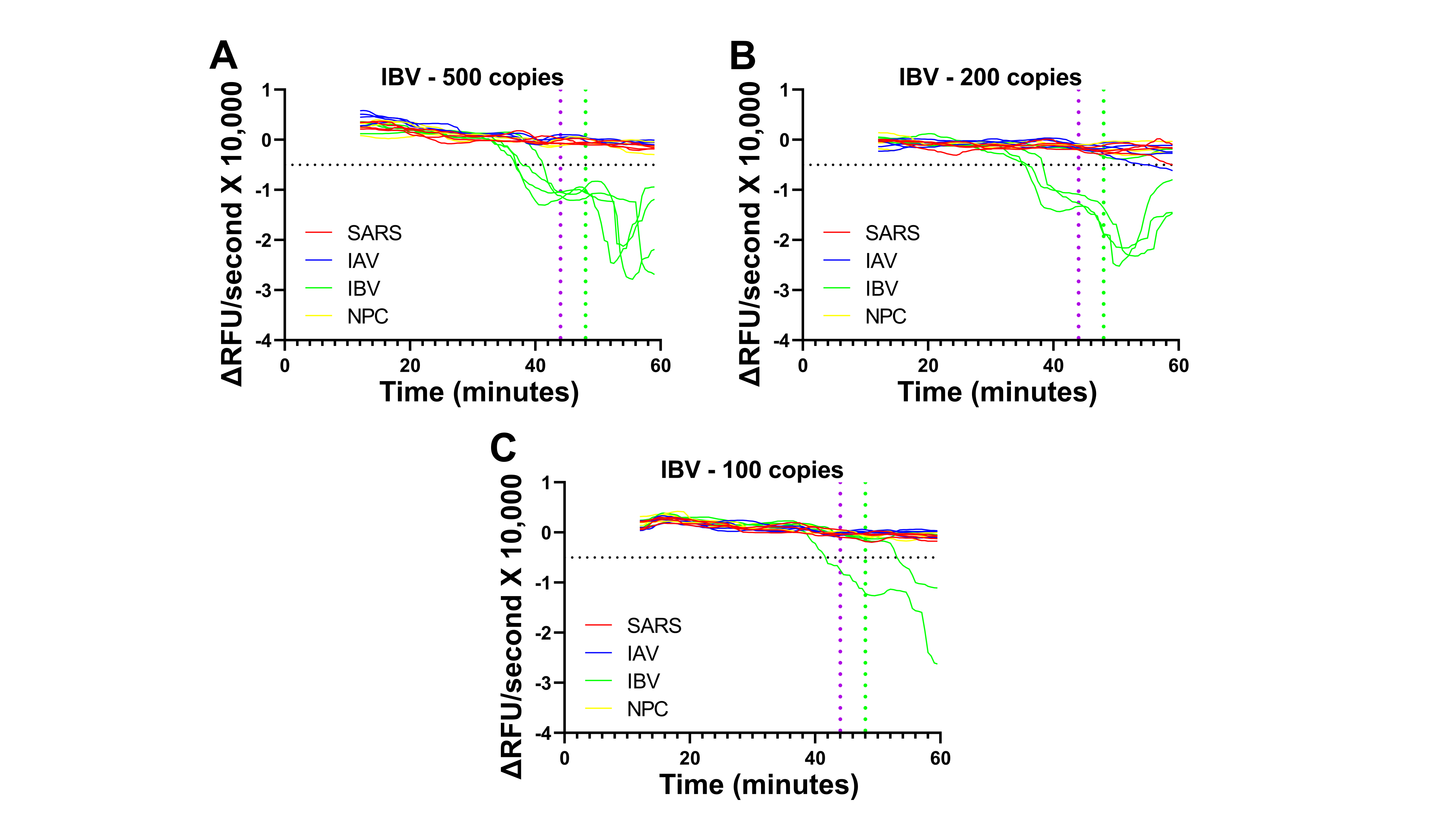


**Supplementary Figure 9:** TVR numerical differentiation of fluorescence curves for assessing influenza B titration outcomes. Numerical derivative plots of fluorescence curves for reactions containing (a) 500, (b) 200, and (c) 100 copies of RNA. Horizontal dotted line indicates threshold for positivity; vertical dotted lines indicate threshold for false positivity of SARS-CoV-2 and IAV reactions (purple) and IBV reactions (green).

**Data for Inactivated Saliva Compatibility Experiments**

RNA controls from IAV, IBV, and SARS-CoV-2 were mixed with inactivated saliva samples to assess their compatibility with RT-LAMP reactions. The resulting normalized fluorescence curves (Supp. Fig. 7) and first derivative curves (Supp. Fig. 8) indicated a diminished sensitivity. Replicate experiments were performed for RT-LAMP experiments without inactivated saliva (Supp. Fig. 9) to enable a statistical comparison between reactions with and without the inactivated saliva.


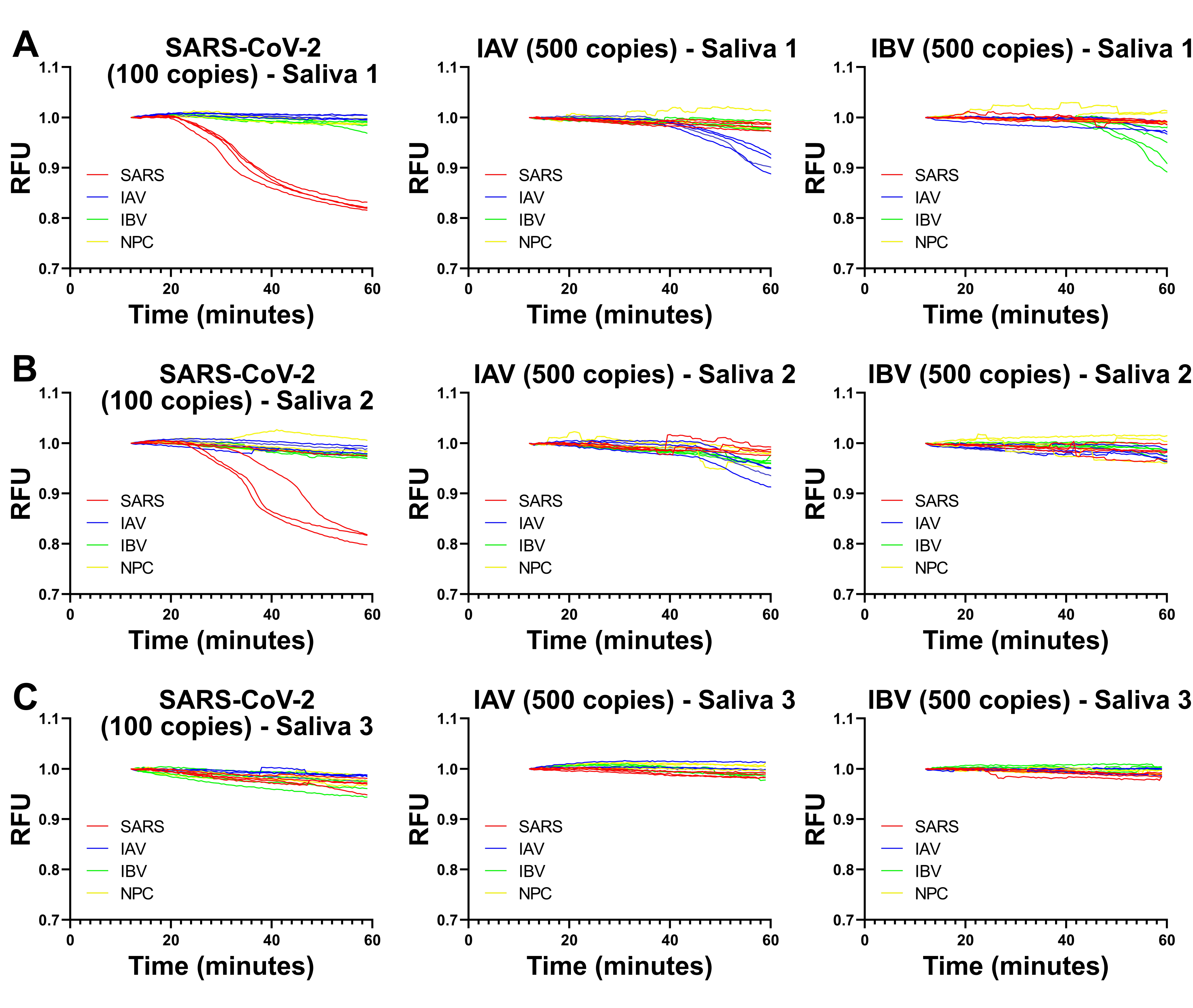


**Supplementary Figure 10:** Inactivated saliva RT-LAMP compatibility fluorescence curves. Relative fluorescence curves for reactions comprising 20% inactivated saliva from (a) donor 1, (b) donor 2, and (c) donor 3 obtained using the indicated number and type of viral RNA copies per reaction chamber.


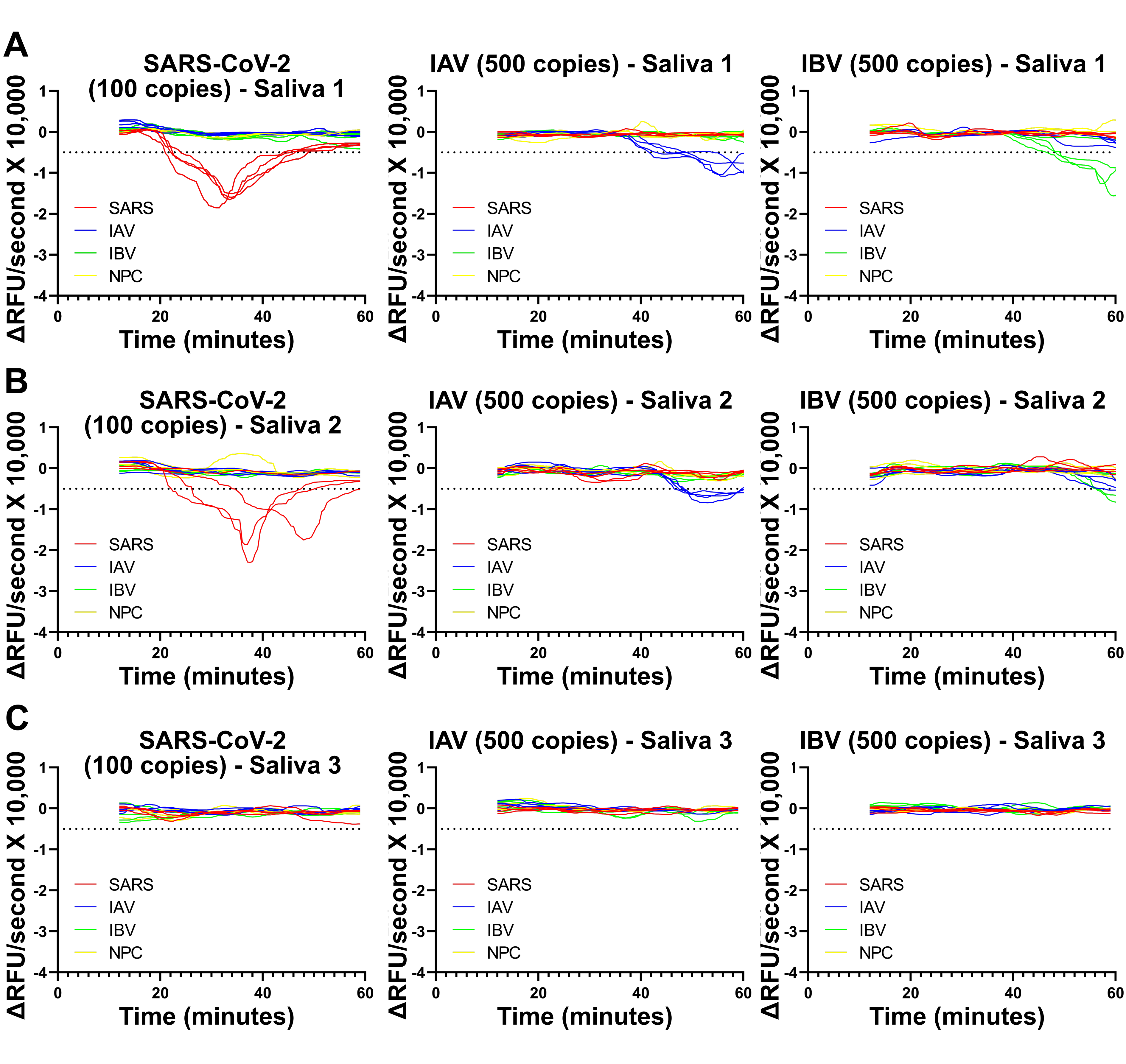


**Supplementary Figure 11:** Numerical derivative analysis to assess inactivated saliva compatibility with RT-LAMP. Numerical derivatives of relative fluorescence curves for reactions comprising 20% inactivated saliva from (a) donor 1, (b) donor 2, and (c) donor 3 obtained using the indicated number and type of viral RNA copies per reaction chamber.


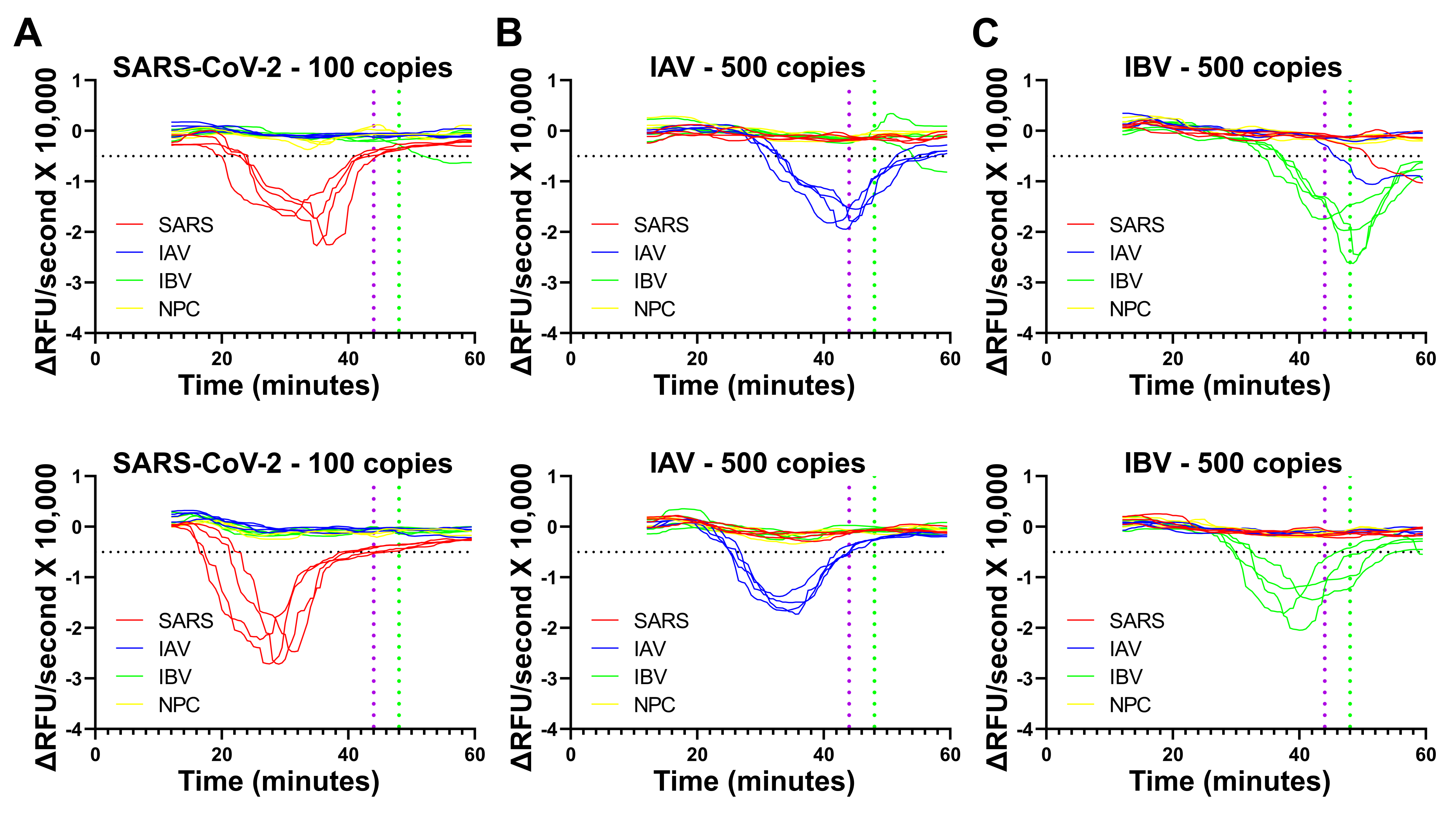


**Supplementary Figure 12:** Numerical derivative analysis of RT-LAMP replicates. Numerical derivatives of relative fluorescence curves for reactions incubated without inactivated saliva and with the indicated copy numbers of (a) SARS-CoV-2, (b) IAV, and (c) IBV RNA.

**Supplementary Table 1:** Cost assessment of services, equipment, materials and reagents for injection molded centrifugal microfluidic chips.

| **All costs are given in USD* | **Commercial molding services** | | | **Desktop Injection Molding** | |
| --- | --- | --- | --- | --- | --- |
|  | **Alpine Mold Engineering Ltd. (China)** | **Dongguan LiangYu Polymer Technology Co., Ltd. (China)** | **Shenzhen Mastech Precision Electronics Co., Ltd. (China)** |  |  |
| **Equipment costs** | ***N/A*** | | | ***Machinery and accessories*** | |
|  |  |  |  | HJK-12T Injection Molder (Haijiang, China) | 4500.00 |
|  |  |  |  | CNC4030-2.2kW milling machine (Jingyan Instruments, China) | 1438.25 |
|  |  |  |  | Miscellaneous components (i.e., metal ceramic heaters, power supplies, pins, springs, end mills etc.) | 166.56 |
|  |  |  |  | **Total** | **6104.81** |
| **Mold costs** | ***Mold fees*** | | | ***Materials and Reagents*** | |
|  | **1918.12** | **3231.00** | **2061.13** | Aluminum stock (milled into A/B mold plates, ejector plates, retaining plate) | 19.20 |
|  |  |  |  | Ejector pins | 4.11 |
|  |  |  |  | **Total** | **23.31** |
| **Cost per molded microfluidic half** | **0.13** | **0.22** | **0.39** | Shot weight: 4.2g; PG-383: USD 1.94/kg | **0.01** |
| **Cost of base for microfluidic chip** | **N/A** | | | 11 cm² of PS sheet and 93005LE PSA | **0.08** |
| **Cost of reagents (per 10 chips)** | **N/A** | | | FITC-pHEMA optical pH sensor | 0.80 |
|  |  |  |  | Sodium polyacrylate | 0.01 |
|  |  |  |  | LAMP primers | 6.60 |
|  |  |  |  | pH calibration buffers | 42.00 |
| **Total costs** | **N/A** | | | **Total reagent cost per chip** | **4.94** |
|  |  |  |  | **Grand total cost per chip** | **5.03** |

**Supplementary Table 2:** Sequences of primers used in this study for RT-LAMP.

| Primer Name | Primer Sequence (5'-3') |
| --- | --- |
| SARS_F3 | CGGTGGACAAATTGTCAC |
| SARS_B3 | CTTCTCTGGATTTAACACACTT |
| SARS_LF | TTACAAGCTTAAAGAATGTCTGAACACT |
| SARS_LB | TTGAATTTAGGTGAAACATTTGTCACG |
| SARS_FIP | TCAGCACACAAAGCCAAAAATTTATTTTTCTGTGCAAAGGAAATTAAGGAG |
| SARS_BIP | TATTGGTGGAGCTAAACTTAAAGCCTTTTCTGTACAATCCCTTTGAGTG |
| IAV_F3 | TTCTAACCGAGGTCGAAAC |
| IAV_B3 | GGACAAAGCGTCTACGC |
| IAV_LF | TCTGCGCGATCTCGGCT |
| IAV_FIP | TGTTCTTTCCTGCAAAGACATTTTTCTATCATCCCGTCAG |
| IAV_BIP | CTAAAGACAAGACCAATCTTTTACTGGGCACGGTGAGCG |
| IBV_F3 | CAGGAAGAGTGGAGCACACTGAAGA |
| IBV_B3 | GATTCGCAAGGCCCTGCT |
| IBV_LF | CTACAGGCACATTCTATGGTT |
| IBV_LB | ATAAGATTGATGTGCACA |
| IBV_FIP | AGGGTCTTTTTGCTGTGTAACTGTTTTTTGCACATGCGGGTTCGCCAG |
| IBV_BIP | GTGGAAACTGATACAGCTGAATTTTTGCTTCCATCATCCGGTCTGG |

**Supplementary References**

1. Cojocaru R, Yaseen I, Unrau PJ, Lowe CF, Ritchie G, Romney MG, Sin DD, Gill S, Slyadnev M (2021) Microchip RT-PCR Detection of Nasopharyngeal SARS-CoV-2 Samples. J Mol Diagnostics 23:683–690. https://doi.org/10.1016/j.jmoldx.2021.02.009

2. Becherer L, Bakheit M, Frischmann S, Stinco S, Borst N, Zengerle R, von Stetten F (2018) Simplified Real-Time Multiplex Detection of Loop-Mediated Isothermal Amplification Using Novel Mediator Displacement Probes with Universal Reporters. Anal Chem 90:4741–4748. https://doi.org/10.1021/acs.analchem.7b05371

3. Gunay M, Goceri E, Balasubramaniyan R (2016) Machine Learning for Optimum CT-Prediction for qPCR. In: 2016 15th IEEE International Conference on Machine Learning and Applications (ICMLA). IEEE, pp 588–592

4. Spata MO, Castagna ME, Conoci S (2015) Image data analysis in qPCR: A method for smart analysis of DNA amplification. Sens Bio-Sensing Res 6:79–84. https://doi.org/10.1016/j.sbsr.2015.10.006

5. Luu-The V, Paquet N, Calvo E, Cumps J (2005) Improved Real-Time RT-PCR Method for High-Throughput Measurements using Second Derivative Calculation and Double Correction. Biotechniques 38:287–293. https://doi.org/10.2144/05382RR05

6. Van Breugel F Van, Kutz JN, Brunton BW (2020) Numerical Differentiation of Noisy Data: A Unifying Multi-Objective Optimization Framework. IEEE Access 8:196865–196877. https://doi.org/10.1109/ACCESS.2020.3034077

7. Chartrand R (2011) Numerical Differentiation of Noisy, Nonsmooth Data. ISRN Appl Math 2011:1–11. https://doi.org/10.5402/2011/164564

8. Oh SJ, Park BH, Choi G, Seo JH, Jung JH, Choi JS, Kim DH, Seo TS, Kim DH, Seo TS (2016) Fully automated and colorimetric foodborne pathogen detection on an integrated centrifugal microfluidic device. Lab Chip 16:1917–1926. https://doi.org/10.1039/c6lc00326e

9. Oh SJ, Seo TS (2019) Combination of a centrifugal microfluidic device with a solution-loading cartridge for fully automatic molecular diagnostics. Analyst 144:5766–5774. https://doi.org/10.1039/C9AN00900K
